# Genomic profiling of human vascular cells identifies *TWIST1* as a causal gene for common vascular diseases

**DOI:** 10.1371/journal.pgen.1008538

**Published:** 2020-01-09

**Authors:** Sylvia T. Nurnberg, Marie A. Guerraty, Robert C. Wirka, H. Shanker Rao, Milos Pjanic, Scott Norton, Felipe Serrano, Ljubica Perisic, Susannah Elwyn, John Pluta, Wei Zhao, Stephanie Testa, YoSon Park, Trieu Nguyen, Yi-An Ko, Ting Wang, Ulf Hedin, Sanjay Sinha, Yoseph Barash, Christopher D. Brown, Thomas Quertermous, Daniel J. Rader

**Affiliations:** 1 Department of Medicine, Perelman School of Medicine, University of Pennsylvania, Philadelphia, Pennsylvania, United States of America; 2 Department of Medicine, Stanford University School of Medicine, Stanford, California, United States of America; 3 Department of Genetics, Perelman School of Medicine, University of Pennsylvania, Philadelphia, Pennsylvania, United States of America; 4 Department of Medicine, Division of Cardiovascular Medicine, University of Cambridge, Cambridge, United Kingdom; 5 Department of Molecular Medicine and Surgery, Karolinska Institute, Solna, Sweden; Brigham & Women’s Hospital and Harvard Medical School, UNITED STATES

## Abstract

Genome-wide association studies have identified multiple novel genomic loci associated with vascular diseases. Many of these loci are common non-coding variants that affect the expression of disease-relevant genes within coronary vascular cells. To identify such genes on a genome-wide level, we performed deep transcriptomic analysis of genotyped primary human coronary artery smooth muscle cells (HCASMCs) and coronary endothelial cells (HCAECs) from the same subjects, including splicing Quantitative Trait Loci (sQTL), allele-specific expression (ASE), and colocalization analyses. We identified sQTLs for *TARS2*, *YAP1*, *CFDP1*, and *STAT6* in HCASMCs and HCAECs, and 233 ASE genes, a subset of which are also GTEx eGenes in arterial tissues. Colocalization of GWAS association signals for coronary artery disease (CAD), migraine, stroke and abdominal aortic aneurysm with GTEx eGenes in aorta, coronary artery and tibial artery discovered novel candidate risk genes for these diseases. At the CAD and stroke locus tagged by rs2107595 we demonstrate colocalization with expression of the proximal gene *TWIST1*. We show that disrupting the rs2107595 locus alters *TWIST1* expression and that the risk allele has increased binding of the NOTCH signaling protein RBPJ. Finally, we provide data that *TWIST1* expression influences vascular SMC phenotypes, including proliferation and calcification, as a potential mechanism supporting a role for *TWIST1* in CAD.

## Author summary

Genome-wide association studies (GWAS) have identified hundreds of genetic variants that are associated with human vascular disease including coronary artery disease. These are predominantly common single nucleotide polymorphisms (SNPs) in non-coding regions, which makes the identification of the causal genes and their underlying connection to pathophysiology challenging. Mapping of expression quantitative trait loci (eQTLs) has been performed to associate GWAS SNPs with risk genes in vascular cells and tissues. However, atherosclerotic vascular tissues contain multiple cell types. We perform deep transcriptomic profiling of genotyped human-derived vascular cells–endothelial cells and smooth muscle cells–and use splicing quantitative trait locus, allele-specific expression, and colocalization analyses to annotate genetic variants associated with vascular diseases and gain insight into their potential function in a cell-type specific manner. Based on these analyses, we identified computationally and then validate experimentally an association between the CAD risk locus rs2107595 and the gene *TWIST1*. We propose that the minor allele for this locus can affect transcription factor binding and provide data supporting a role for *TWIST1* in modulating smooth muscle cell phenotype.

## Introduction

Coronary artery disease (CAD) is a complex vascular wall process characterized by progressive development of atherosclerotic plaques involving multiple cell types. Family and twin studies estimate the heritability of risk for CAD at 40% to 60% [1]. Genome-wide Association Study (GWAS) meta-analyses have reported more than 160 genomic loci that are significantly associated with CAD [2–5]. A subset of these loci are also associated with other phenotypes, including lipid traits [6], hypertension, stroke [7], migraine [8], and abdominal aortic aneurysm [9]. The identified GWAS variants are predominantly common single nucleotide polymorphisms (SNPs) in non-coding regions, which makes the identification of the causal genes and their underlying connection to pathophysiology challenging. Mapping of expression quantitative trait loci (eQTLs) has been performed to associate GWAS SNPs with risk genes in vascular cells and tissues [10–12]. However, atherosclerotic vascular tissues contain multiple cell types. Cell-specific analysis of eQTLs would considerably advance our understanding of the underlying biology.

We therefore performed transcriptomic analysis and genome-wide genotyping in 19 paired primary human coronary artery smooth muscle cell (HCASMC) and coronary artery endothelial cell (HCAEC) lines. Using this resource, we performed splicing quantitative trait loci (sQTL) and allele-specific expression (ASE) analyses to identify and stratify vascular disease risk genes in these two major cell types of the coronary vessel wall. Additionally, we performed colocalization analysis between GWAS signals for four vascular traits (CAD, stroke, migraine, abdominal aortic aneurysm) and eQTL regulated Genes (“eGenes”) in three arterial GTEx tissues (aorta, coronary artery, tibial artery) to identify regulated, disease-relevant genes. As an example of the potential of this approach, we show a previously unreported association of variants at the rs2107595 CAD GWAS locus with expression of the *TWIST1* gene. We then demonstrate that the rs2107595 locus can regulate *TWIST1* expression and provide evidence for a functional role of *TWIST1* in vascular smooth muscle cells.

## Results

### Cultured primary vascular cells maintain expression features of primary tissue

**Fig 1:**
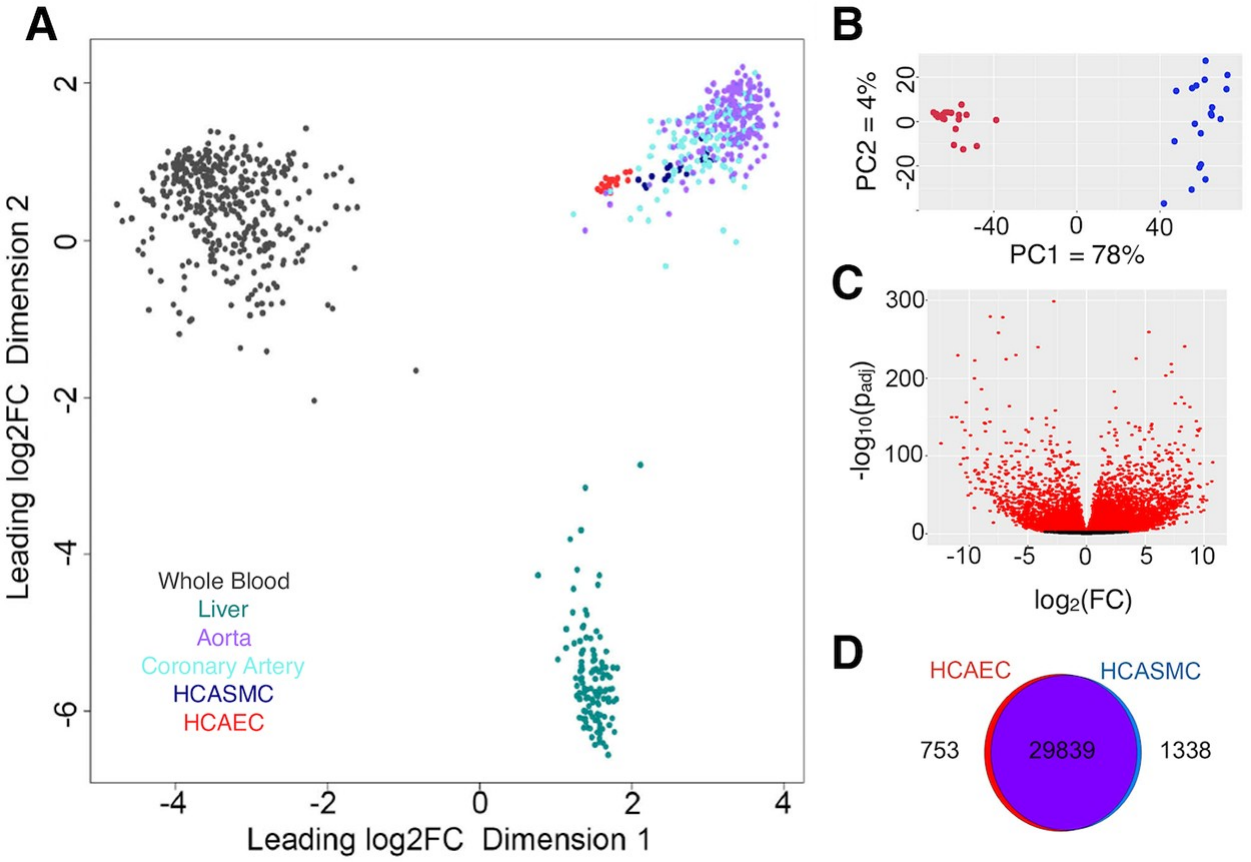
Transcriptional profiling of primary HCASMCs and HCAECs. A) Multi-dimensional scaling plot (500 most differentially expressed genes from RNASeq data from both GTEx–whole blood, liver, aortic and coronary artery samples–and HCASMC and HCAEC samples) highlights that *in vitro* cultured primary cells cluster with vascular tissues and HCASMCs displays a bigger overlap with the primary tissue. B) Principle component analysis of *in vitro* cultured coronary artery cells showing 78 percent of the expression variability can be explained by cell type (PC1 –x-axis). Endothelial cells show less transcriptional diversity than the smooth muscle cells (PC2 –y-axis). HCAECs in red, HCASMCs in blue. C) Volcano plot of differentially expressed genes distributed by log2 fold change (x-axis) and adjusted p-value (y-axis). Genes with a positive fold change are overexpressed in HCASMCs relative to HCAECs. Genes in red meet the significance threshold of adjusted p-value ≤0.001. D) Venn diagram of genes differentially expressed in HCASMC and HCAEC at adjusted p-value≤0.001 and log2(FoldChange) ≤ -2 or ≥2.

We deep-sequenced the transcriptomes of 19 donor-matched pairs of cultured human coronary artery smooth muscle (HCASMCs) and endothelial cells (HCAECs) (S1 Table). Together with available transcriptome data from human liver, whole blood, aorta and coronary artery from the Genotype-Tissue Expression (GTEx) project [13, 14], these were aligned to the human genome (Ensembl v90, hg19) and analyzed for differential gene expression. Upon stringent quality control measures, a total of 15 samples with genotype, HCASMC RNA-seq, and HCAEC RNA-seq were retained for ASE and sQTL analyses. Multi-dimensional scaling of gene expression data show how both HCASMC and HCAEC samples cluster relative to arterial tissues when compared with liver or whole blood (Fig 1A), and with each other when compared solely with arterial tissue (S1B Fig). Principal component analysis demonstrates that cell type explains 78 percent of the observed variance, with the second principal component explaining 4 percent (Fig 1B).

Differential gene expression analysis identified 2091 differentially expressed genes between the two coronary cell types (Fig 1C), with 753 (HCAEC) and 1,338 (HCASMC) genes meeting strict filtering criteria (log2 (fold change) ≥2, adjusted p-value ≤ 0.001, Fig 1D, S2 File). Genes preferentially expressed in HCASMCs were from lineage-specific pathways such as epithelial-to-mesenchymal transition, myogenesis, angiogenesis and hepatic fibrosis (Table 1); HCAEC-specific genes were in Notch, eNOS and VEGF signaling pathways (S2 Table).

**Table 1:** Pathway analysis of genes overexpressed in HCASMCs.

**GSEA Name**	**Normalized Enrichment Score**
HALLMARK_EPITHELIAL_MESENCHYMAL_TRANSITION	2.391177
HALLMARK_MYOGENESIS	1.95380
HALLMARK_ANGIOGENESIS	1.57199
HALLMARK_COAGULATION	1.555932
HALLMARK_HYPOXIA	1.554287
HALLMARK_UV_RESPONSE_DN	1.535091
HALLMARK_GLYCOLYSIS	1.524811
HALLMARK_ESTROGEN_RESPONSE_EARLY	1.509326
HALLMARK_TNFA_SIGNALING_VIA_NFKB	1.469919
HALLMARK_ESTROGEN_RESPONSE_LATE	1.428466
**Ingenuity Canonical Pathways**	**Adjusted P-Value**
Hepatic Fibrosis / Hepatic Stellate Cell Activation	5.47E-19
Granulocyte Adhesion and Diapedesis	8.85E-05
Axonal Guidance Signaling	1.30E-04
Regulation of the Epithelial-Mesenchymal Transition Pathway	2.83E-04
Agrin Interactions at Neuromuscular Junction	2.83E-04
Acute Phase Response Signaling	2.83E-04

### Splicing quantitative trait locus (sQTL) analysis suggests potential mechanisms underlying vascular GWAS findings

To examine the effect of GWAS loci for vascular disease on the relative abundance of RNA splice isoforms, we performed a genome-wide screen for sQTLs in HCAECs and HCASMCs. Using MAJIQ, which quantifies local splicing variations (LSVs) as percent spliced in (PSI) of alternatively-spliced mRNA segments, we identified 478 SNPs in 196 genes (SMC) and 1028 SNPs in 359 genes (EC) which were nominally associated (p<0.05) and passed 0.05 FDR correction at the gene level (S1 File). Combined, these lists included 1399 unique SNPs in 512 genes. Next, we took 3,844 unique genes with at least one nominally significant sQTL in HCASMC or HCAEC and tested those for sQTL using GTEx artery tissues. Of those, 33057 SNPS in 3310 genes were validated in at least one GTEx tissue at FDR < 0.05. We compared these lists with the set of SNPs and genes with putative sQTLs passing 0.05 FDR correction at the gene level in HCASMC and HCAEC. 924 SNPs in 471 genes were reproduced in GTEx, whereas 475 SNPs in 41 genes were unique to the two cell types (S2A Fig). Finally, we also performed genome wide sQTL analysis for the three artery GTEX tissues and identified 54298 unique SNPs in 7965 genes that passed 0.05 FDR correction at the gene level (S3 File).

The vast majority of GWAS-associated loci for vascular phenotypes have not been functionally annotated. Colocalization analysis combines two different data sets to see if related phenotypes share genetic variants. If a SNP colocalizes between a gene-specific phenotype and a disease phenotype, there is greater confidence that the variant and gene may be causal for the disease. We queried for colocalization between published GWAS loci for vascular disease and the sQTL loci identified above. For sQTL loci identified from HCASMCs and HCAECs, we found one SNP at the *TARS2* gene and 4 SNPs in 3 genes (*YAP1*, *CFDP1*, and *STAT6*) for HCASMCs and HCAECs, respectively, that passed 0.05 FDR correction at the gene level (S3 Table). All of these variants are in linkage disequilibrium (LD > = 0.8) with SNPs which are associated with migraine at genome-wide significance. Of these, rs167769 is both associated with migraine and an sQTL associated with an alternative 5’ splice site in the first exon of five of the six annotated transcripts of *STAT6*. This splicing variation affects the 5’ UTR (sQTL FDR = 0.047) and accounts for approximately 7% increase in inclusion of the 18-nt extension (S3 Table and S3 Fig).

From the GTEx genome-wide sQTL analysis for arterial tissues described above, we identified 20 SNPs in 5 genes in aorta, 10 SNPs in 6 genes in coronary artery, and 29 SNPs in 8 genes in tibial artery achieved genome wide significance for association with their respective diseases (S2B Fig, S3 Table). Of note, rs324011 is a significant sQTL for *STAT6* in all three GTEx tissue types (sQTL FDR = 0.00351 in aorta, 0.00349 in coronary artery, and 0.00271 in tibial artery). This variant is in strong LD with rs167769 discussed above (LD score = 0.943414), which associates with the same splicing variation in HCAEC but also in aorta and tibial artery (S3 Fig). In addition, rs324011 was identified as a nominal eQTL for *STAT6* in all three GTEx tissue types (eQTL p = 0.00139 in aorta, 0.0453 in coronary artery, and 4.17x10^-5^ in tibial artery) (S3 Table). This overlap between sQTL and eQTL was observed for several of the aforementioned SNPs (see complete table of sQTL and eQTL overlap in S3 File) and may point to mechanistic connections such as splicing induced frameshifts that lead to nonsense mediated decay and result in decreased gene expression.

Finally, to identify which of the identified sQTLs may be causal we assessed the associated SNPs for predicted effect on splicing using ENSEMBL’s Variant Effect Prediction (VEP) tool [15]. Of the 26769 putative sQTLs called with FDR < 0.05 in any GTEx artery tissue (Aorta, Coronary artery, or Tibial artery), 488 SNPs in 400 genes lay within range of annotated splice sites to be scored by MaxEntScan tool (S3 File) [16]. While none of these overlapped with the sQTLs which were also cardiovascular disease-associated described above, 59 variants were predicted to have a high impact on splicing (diff > = 1.15 and alt < 6.2 OR diff < = -1.15 and alt > 8.5, see [4]). The strongest predicted effect is that of rs3762374 (G → A), an intronic variant located five positions past the 3’ end of *DRAM2* cassette exon 2. This variant was previously flagged as a QTL for both splicing and expression of *DRAM2* Lalonde et al 2012], and the alternative A allele associates with decreased inclusion of the cassette exon in all three GTEx artery tissue types (FDR = 7.01x10^-36^, 3.20x10^-13^, and 1.04x10^-40^ in aorta, coronary artery, and tibial artery, respectively) (S3 Table), in line with the directionality of the MaxEntScan prediction. This splicing variation affects the 5’ UTR of the two annotated protein-coding transcripts and may affect translation initiation efficiency.

### Allele-specific expression occurs predominantly in arterial eGenes

**Fig 2:**
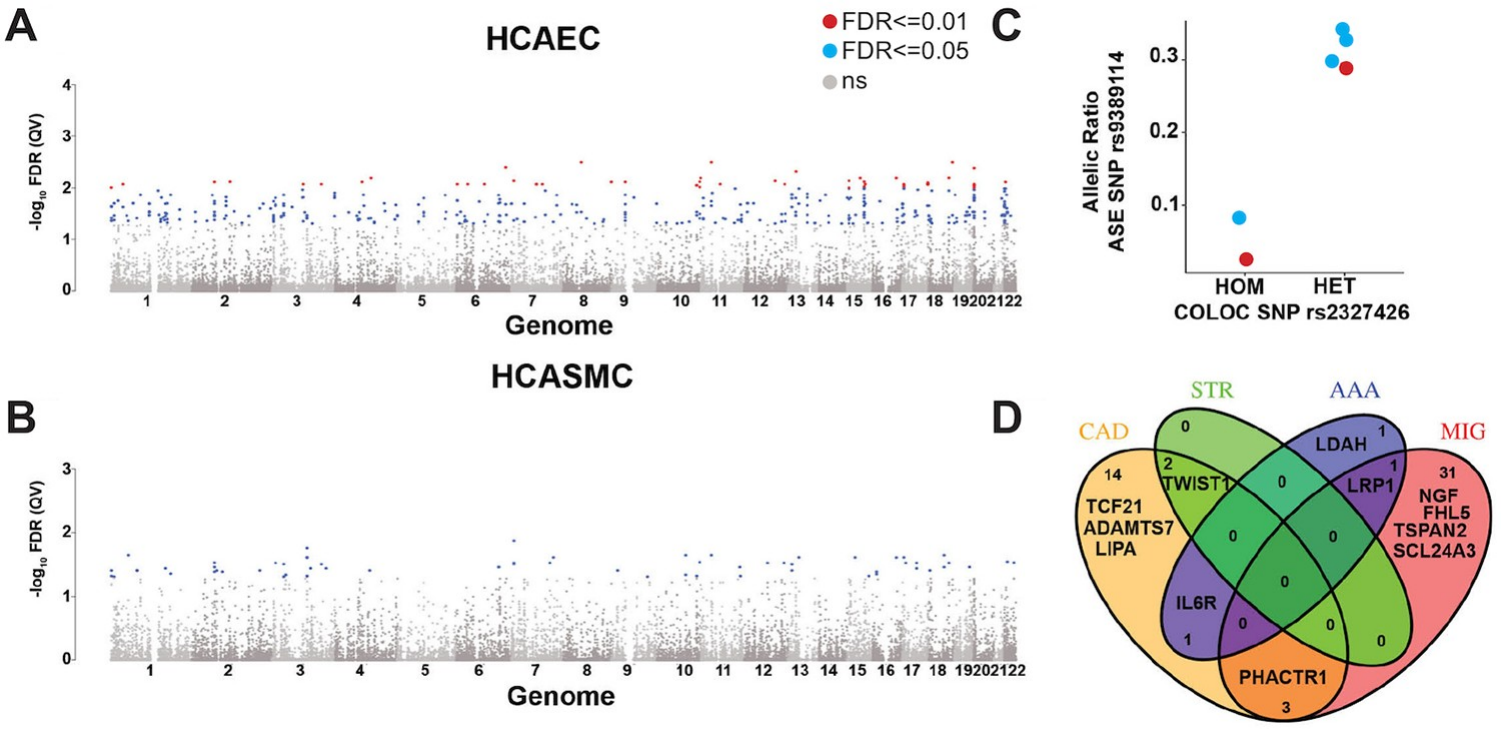
Non-coding variation modulating expression in vascular cells and tissues. A) and B) Manhattan plot of Allele-Specific Expression loci in HCAECs (A) and HCASMCs (B). Genomic coordinates (hg19) are plotted on the x-axis, Benjamini-Hochberg adjusted p-values (FDR) for each interrogated variant are plotted on the y-axis. SNPs with FDR ≤ 0.05 are shown in blue and those with FDR ≤ 0.01 in red. C) Allele-Specific Expression of TCF21 based on colocalization genotype. Allelic expression ratio (A / (A+R) = 0.5) of TCF21 at rs9389114 on the y-axis is associated with genotype of the lead colocalization variant rs2327426 on the y-axis. HCAECs are shown in red and HCASMCs in blue. D) Venn diagram of colocalization genes for 4 vascular traits. Coronary Artery Disease (CAD), Stroke (STR), Abdominal Aortic Aneurysm (AAA) and Migraine (MIG). Numbers in sectors represent the numbers of colocalization genes per group. HGNC gene names of key association genes are plotted as well.

To identify genetic variants that affect gene expression in cis, we quantified ASE using QuASAR at FDR threshold of 0.05. We observed ASE at 342 SNPs, corresponding to 206 genes in HCAECs (Fig 2A) and at 63 SNPs, corresponding to 48 genes in HCASMCs (Fig 2B). ASE was shared across cell types at 27 SNPs (21 genes) (S4 Table). Comparison of these genes with GTEx data showed many of the ASE genes we identified were also expression quantitative trait loci (eQTLs) in arterial tissues in GTEx; we refer to these as eGenes. In fact, the majority of these eGenes overlap between aorta, coronary artery, and tibial artery where 205 of 233 ASE genes (88%) were also eGenes in all three arterial tissues. Only myosin heavy chain 13 (*MYH13*), which displayed ASE selectively in HCASMCs, was a GTEx eGene specifically in coronary artery and not the other arterial tissues.

### Colocalization analysis of arterial tissue eGenes and vascular disease-associated GWAS loci identifies new candidate risk genes

As with sQTL loci, we hypothesized that colocalization analysis of GWAS loci for vascular disease and eQTLs in arterial tissues may help disentangle gene-rich loci and implicate novel candidate genes. We found colocalization with a locus-wide posterior probability threshold of 0.7 (PP.H4.abf) in 93 variants at 53 gene loci (S5 Table). Of the 53 colocalization genes, *TCF21*, *MFGE8* and *UFL1* also showed ASE in cultured vascular cells. *MFGE8* (milk fat globule-EGF factor 8 protein) is a novel risk gene for CAD and encodes lactadherin and its cleavage product medin, which comprises the major protein component of aortic medial amyloid (AMA) [17–19]. *UFL1* (UFM1 specific ligase 1) is a novel migraine risk gene involved in ufmylation, a post-translational modification on lysine residues of proteins, that may play a crucial role in a number of cellular processes. Its role in migraine is still largely unknown [20]. *TCF21* is a CAD risk gene with a well-established role in HCASMCs [21–23]. Specifically for *TCF21*, allele-specific expression of either rs9399114 or rs9402547 (r^2^ = 1) was correlated with genotype at the colocalization lead SNP rs2327426 (p-value = 0.1336, Wilcoxon rank sum test) (Fig 2C, S4A Fig).

GTEx eQTL signals colocalized with CAD-associated variants in 32 cases (20 genes), stroke-associated variants in 2 cases (2 genes), abdominal aortic aneurysm (AAA)-associated variants in 6 cases (3 genes), and migraine-associated variants in 53 cases (35 genes) (S6 Table). We identified colocalization signals at several established single trait loci such as *TCF21*, *ADAMTS7*, and *LIPA* for CAD, and *NGF*, *FHL5*, *TSPAN2*, and *SLC24A3* for stroke. Gene-rich association loci such as *VAMP5-VAMP8-GGCX* (CAD) and *LRP1-STAT6-SDR9C7* (migraine) colocalized only with *GGCX* and *LRP1* in arterial tissues. Some associations co-occurred in 2 traits (Fig 2D), namely for CAD and stroke at *TWIST1*, for CAD and AAA at *IL6R*, for CAD and migraine at *PHACTR1*, and for migraine and AAA at *LRP1*. *LRP1* and *IL6R* play well-described roles in vascular biology and disease [24, 25]. The association between PHACTR1 loci and vascular diseases is an ongoing area of investigation [26], and PHACTR1 locus rs934937 has been linked to both endothelin1 and PHACTR1 genes [27, 28]. However, little is known about the role of *TWIST1* in vascular diseases.

**Fig 3:**
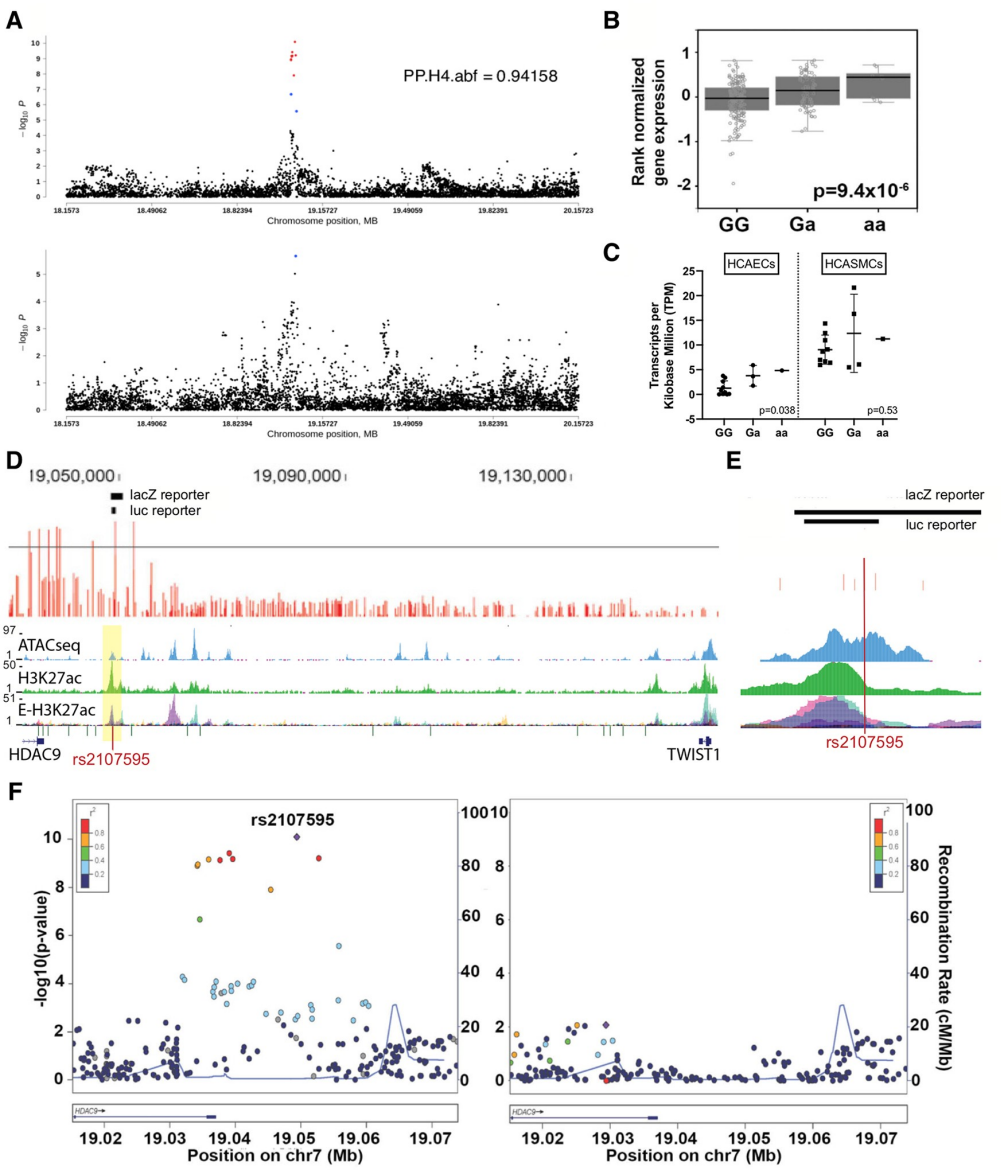
The TWIST1/HDAC9 association locus for CAD and stroke. A) Colocalization analysis of GWAS association with CAD (upper panel) and eQTLs for expression of TWIST1 in human aorta (lower panel). The position on Chromosome 7 (hg19) is plotted on the x-axis, -log10 adjusted P-value on the y-axis. The locus-wide posterior probability that both the GWAS association and the eQTL association are based on the same SNP(s) (PP.H4.abf) is 0.94158. B) TWIST1 expression in human aorta based on rs2107595 genotype (GTEx v7 data, n = 267). C) TWIST1 expression in HCAECs (n = 14) and HCASMCs (n = 14) based on rs2107595 genotype. D) Genomic locus at the rs2107595 association signal for Coronary Artery Disease (CAD). The upper panel in red represents SNPs tested for association with CAD. The line demarcates the p-value threshold of genome-wide significance at 5x10^-8^. Above are depicted the regions assayed in downstream analyses. The panels below the gene annotation track show regions of potentially functional chromatin including regions of open chromatic (ATACseq) in HCASMC, enhancers (H3K27ac) as identified in HCASMC and enhancers identified in multiple ENCODE tissues (E-H3K27ac). E) Close-up of the rs2107595 region. At the top are shown the region tested in vivo for enhancer function in mouse embryo and below in luciferase reporter assays. The lead association SNP falls into a putative functional region. F) Conditional analysis at the rs2107595 association locus for CAD before (left) and after (right) association for linkage. Plotted are all tested SNPs along the genomic coordinate at the locus (x-axis) and their p-value of significance of association for CAD (left y-axis). The right y-axis shows the recombination rate at the locus as a measure of linkage disequilibrium.

We further investigated cases in which colocalization eGenes differed from reported annotated genes at GWAS loci. For example, the reported migraine-associated signal near calcium responsive transcription factor (*CARF*) displayed colocalization with neurobeachin like 1 (*NBEAL1*) expression in all three arterial tissues. Rare protein coding variants at *NBEAL1* were recently demonstrated to be associated with raised atherosclerotic lesions in the young [29]. Importantly, the shared stroke and CAD association signal rs2107595 near *HDAC9* colocalized with *TWIST1* but not *HDAC9* expression in aorta (Fig 3A). In additional to our colocalization results, the CAD GWAS lead SNP rs2107595 is associated with ischemic heart disease and disease of the precerebral arteries in UK Biobank dataset (S6B Fig) [30] and with stroke [31], Moyamoya disease (a rare, progressive cerebrovascular disorder) [32], and peripheral artery disease (PAD) [33]. We therefore focused on the rs2107595 locus for further characterization.

### TWIST1 as novel candidate risk gene for CAD and stroke

Although the lead SNP rs2107595 is shared between CAD (adjusted p-value = 8.05x10^-11^), stroke (large vessel disease, adjusted p-value = 2·03×10^−16^) and Moyamoya disease (adjusted p-value = 1.49x10^-29^), the variant associated with PAD in a Japanese population (rs2074633, adjusted p-value = 8.8x10^−8^) is in high linkage disequilibrium with rs2107595 in the East Asian population (r^2^ = 0.832 (CHB/JPT) / 0.617 (EUR)– 1000G Phase I data). The association signal is located near the last exon of the *HDAC9* gene and has therefore been previously reported as an *HDAC9* association locus. However, rs2107595 is a GTEx eQTL locus for *TWIST1* in aorta (Fig 3B, S4B Fig), and our colocalization analysis showed that this region colocalizes with TWIST1 (Fig 3A).

In our transcriptome data, although *TWIST1* was associated with rs2107595 genotype in HCAECs but not in HCASMCs (Fig 3C), it was preferentially expressed in HCASMCs versus HCAECs (S5A Fig). Furthermore, in human carotid plaque samples *TWIST1* expression was positively correlated with SMC markers in human (S5B Fig). In total, four of the SNPs reaching genome-wide significance for association with CAD in this locus fall into known enhancers or transcription factor binding sites in HCASMCs. rs2107595 lies in an Assay for Transposase-Accessible Chromatin using sequencing (ATACseq) site in human aortic smooth muscle cells as well as an H3K27Ac enhancer in HCASMCs (Fig 3D and 3E) and an H3K27ac site in mesenchymal stem cells (S6 Fig). Finally, conditional analysis of the association in this region with CAD indicates that the rs2107595 haplotype is the primary major determinant of the observed association (Fig 3F).

**Fig 4:**
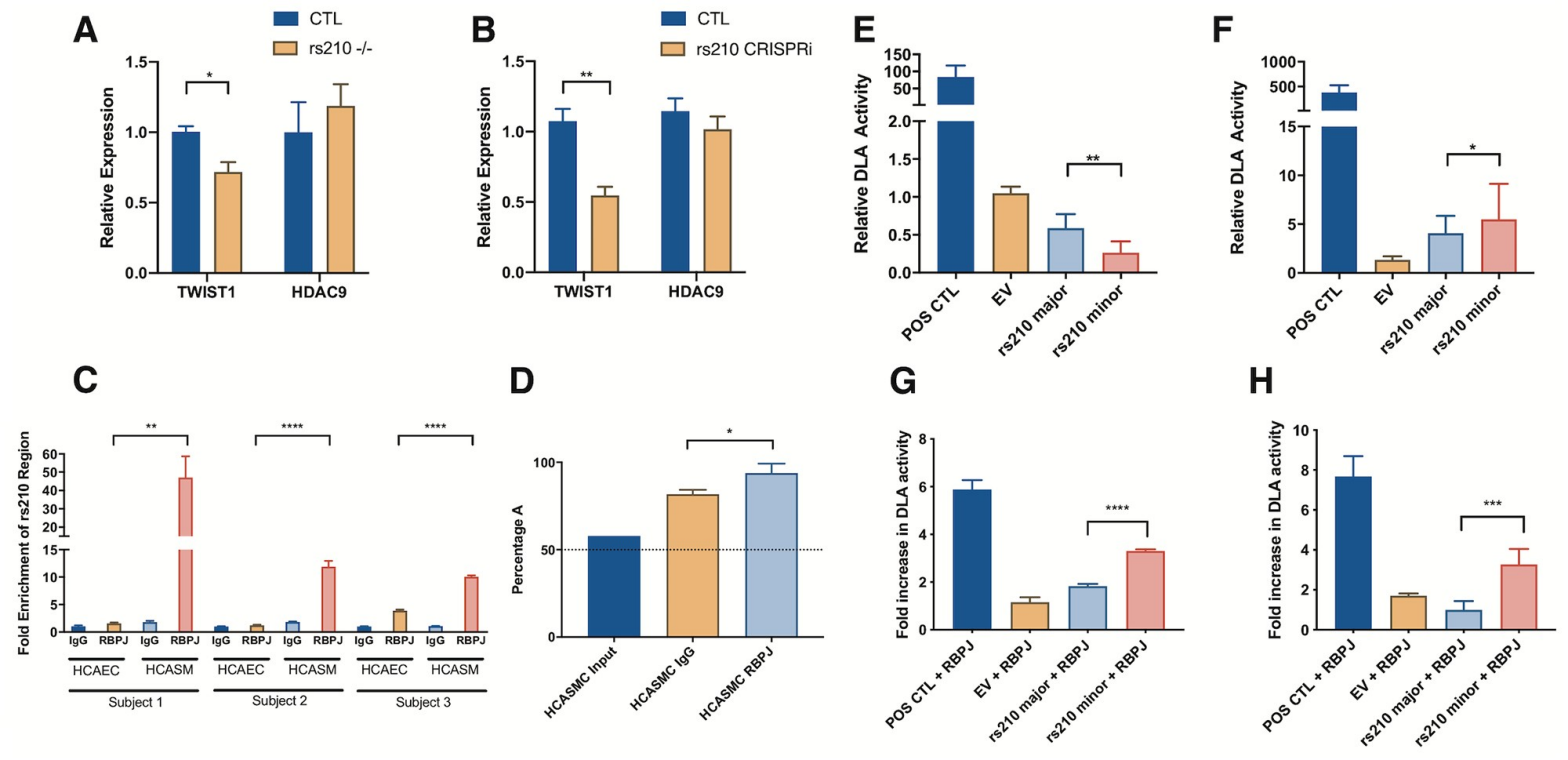
Functional annotation of the rs2107595 region in HEK293T, A7r5, and HCASMCs. *p<0.05, ** p< 0.01, ***p<0.001, ****p<0.0001. A) Clonal HEK293T colonies with disruption of rs2107595 locus with CRISPR genome editing show decreased average *TWIST1* expression and no significant difference in *HDAC9* expression.B) CRISPR inhibition (CRISPRi) targeted to rs2107595 in HCASMCs decreased *TWIST1* expression with no significant effect on expression of *HDAC9*. A single guide directed to the GWAS lead SNP rs2107595 was cloned into a lentiviral plasmid that encodes dCas9-KRAB and transduced into HCASMC. C) ChIP-qPCR in three separate heterozygous HCAEC and HCASMC lines showed increased binding of RBPJ to rs2107595 locus in HCASMC compared to HCAECs. D) Allele-specific analysis of RBPJ ChIP was performed in three separate heterozygous HCASMCs lines. The averaged results shows preferential pull-down of the minor (A, risk) allele of rs2107595. The dashed line marks the 50 percent allelic balance. E, F) The genomic region around rs2107595 serves a regulatory function in both HEK293 cells (repressor) and in A7r5 SMCs (enhancer) in luciferase reporter assays. In both cases, the minor (risk) allele increases the regulatory function of the region. G,H) When compared with the major allele, the minor allele showed increased luciferase activity in both HEK293 (G) and A7r5 (H) cells in transactivation assays with a constitutively active RBPJ-VP16 fusion protein.

To evaluate the effect of rs2107595 on *TWIST1* expression, the rs2107595 locus was edited using CRISPR/Cas9 in HEK293T cells. Following transfection with plasmid containing CRISPR/Cas9 and a guide RNA targeted upstream of variant rs2107595, individual clones were expanded and evaluated by sequencing of genomic DNA. *TWIST1* and *HDAC9* expression were evaluated in each clone with editing at the rs2107595 locus (S7 Fig). Cell lines in which the rs2107595 was disrupted had decreased *TWIST1* expression with no significant effect on HDAC9 (Fig 4A). To evaluate the role of rs2107595 in HCASMCs specifically, a single guide RNA targeting the region at this variant along with a CRISPR-KRAB expression cassette were transduced by lentivirus into HCASMCs. Epigenetic silencing at rs2107595 decreased *TWIST1* expression without affecting HDAC9 expression (Fig 4B).

Based on *in silico* analyses, the major (protective) allele of rs2107595 harbors an E2F binding motif, while the minor (risk) allele generates an RBPJ binding motif (S8 Fig). RBPJ is the major effector of the Notch signaling pathway which is known to be important in vascular development and disease [34, 35], and to transcriptionally regulate *TWIST1* expression [36, 37]. To evaluate RBPJ binding at the rs2107595 locus, we performed chromatin immunoprecipitation for RBPJ in HCASMCs and HCAECs from three heterozygous donors. RBPJ binding at rs2107595 was selectively enriched in HCASMCs relative to HCAECs (Fig 4C). We also confirmed that E2F is able to bind to this region (S9B Fig). Allele-specific analysis demonstrated that RBPJ preferentially binds to the risk allele (Fig 4D, S10 Fig).

We hypothesized that, in contrast to the protective major allele, the risk allele promotes RBPJ binding at this site resulting in increased transcriptional activity. To test this hypothesis, we examined an 800 bp genomic region around rs2107595 for transcriptional regulatory activity using *in vitro* luciferase reporter assays in HEK293T cells and rat aortic smooth muscle (A7r5) cells. The genomic region served as a repressor in HEK293T cells and an enhancer in SMCs, which may be due to differences in cell-specific transcription factors or adaptor proteins [38]. In both cases, the risk allele increased transcriptional activity of the region (Fig 4E and 4F). Transactivation assays with a constitutively active RBP show that in the presence of RBPJ, the major (risk) allele is stimulated to a larger extent than the minor (protective) allele (Fig 4G and 4H).

### TWIST1 plays a functional role in SMCs in vitro

**Fig 5:**
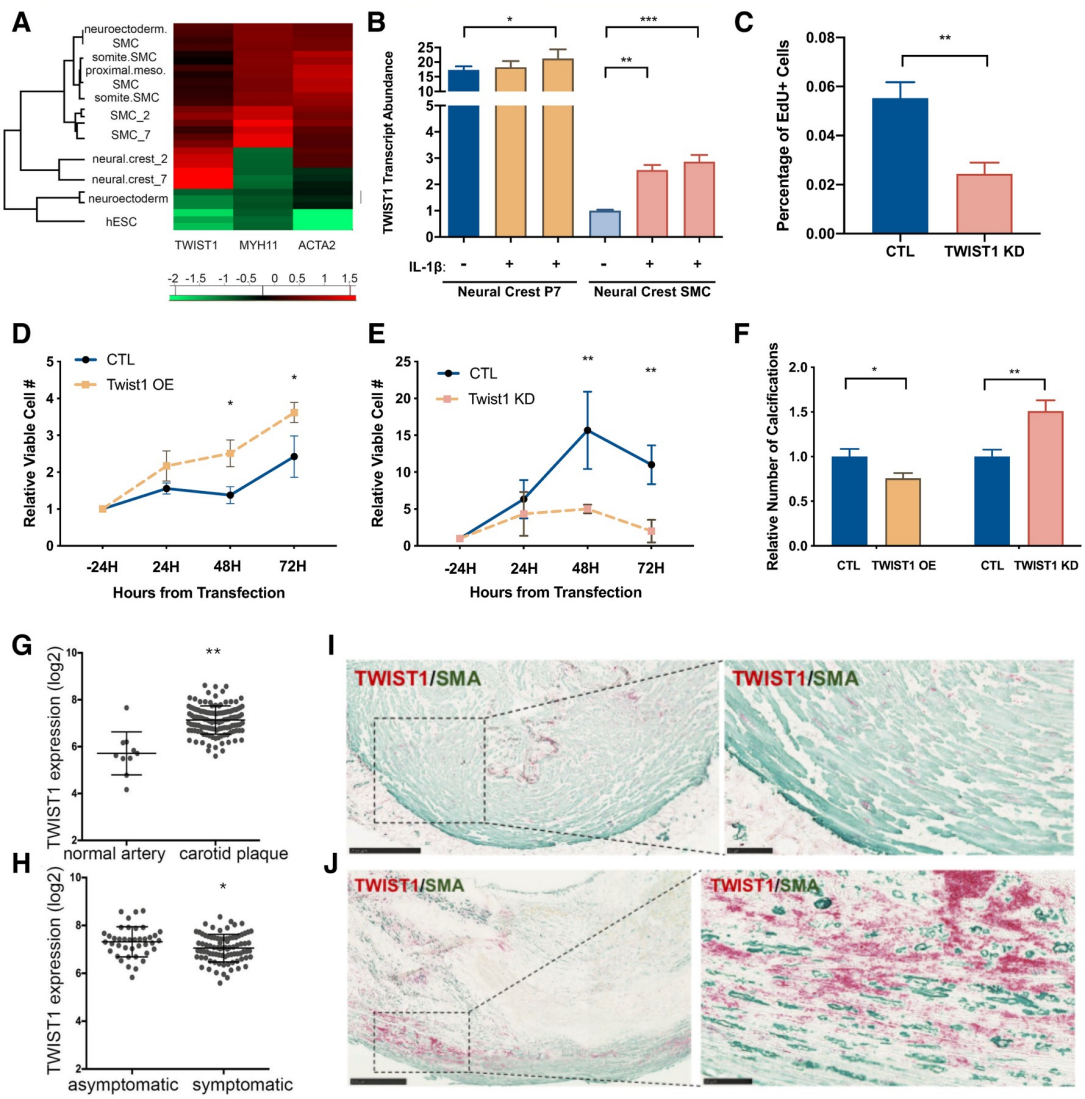
The role of TWIST1 in vascular cells. A) Heat map showing the differential gene expression of TWIST1 and an early stage (ACTA2) and late stage (MYH11) differentiation marker of vascular SMCs in an *in vitro* differentiation model. Values represent Euclidean distances from the mean across all samples at the different stages. During *in vitro* differentiation of SMCs, TWIST1 expression is maximal at the neural crest progenitor stage and decreases during smooth muscle cell differentiation. (hESC: human embryonic stem cells; neuroectoderm: neuroectoderm progenitors; NC_2: neural crest progenitor passage 2; NC_7: neural crest progenitor passage 7; SMC_2 NC: SMCs derived from NC_2; SMC_7: SMCs derived from NC_7; somite.SMC: SMCs derived from paraxial mesoderm progenitor; proximal.meso.SMC: SMCs derived from lateral mesoderm progenitor; neuroectoderm.SMC: SMCs derived from neuroectoderm progenitors). B) Quantitative PCR of TWIST1 expression levels in neural crest (neural crest P7) progenitor cells and neural crest derived smooth muscle cells (neural crest SMC) with and without stimulation with IL-1β. TWIST1 expression is upregulated in neural crest-derived SMCs after treatment with IL-1β. C) EDU assay of HCASMCs showed decreased proliferation with knockdown of TWIST1. D,E) Proliferation assay in A7r5 aortic smooth muscle cells: *TWIST1* overexpression increased cell proliferation significantly at 48H and 72H after transfection, whereas *TWIST1* siRNA knockdown decreased cell proliferation at 48H and 72H. F) Calcification assay in A7r5 aortic smooth muscle cells: *Twist1* overexpression decreases calcification and Twist1 knockdown increased calcification based on alizarin red staining. G)–J) TWIST1 expression in human carotid tissue. *TWIST1* is significantly overexpressed in diseased human carotid endarterectomy samples relative to normal arteries (E), and *TWIST1* expression is higher in asymptomatic plaques relative to symptomatic plaques (F). Immunohistochemistry of normal carotid arteries show that there is low basal TWIST1 expression (red) which colocalizes with smooth-muscle-actin (SMA, green) positive SMCs in the vessel media (H). TWIST1 expression is increased in SMC-rich regions of carotid plaques (H).

To determine a possible role of TWIST1 in vascular disease, we next investigated TWIST1 abundance and function in vascular smooth muscle cells. Using a human pluripotent stem cell derived neural crest model of *in vitro* lineage-specific differentiation of vascular smooth muscle cells [39], we found that *TWIST1* expression was highest at the SMC progenitor stage, particularly in neural crest progenitor cells which selectively contribute to formation of the aortic arch (Fig 5A). *TWIST1* expression was decreased in the differentiated SMCs arising from these progenitors. To determine whether *TWIST1* expression could be induced in differentiated SMCs, and therefore be relevant in adult pathology as well as in development, we stimulated cells with interleukin (IL)-1β, an inflammatory cytokine upregulated during atherosclerosis disease progression. Indeed, *TWIST1* expression was induced by IL-1β (Fig 5B, S11 Fig).

Since *TWIST1* was more highly expressed in SMC progenitor states and was activated by IL-1β stimulation, we hypothesized that TWIST1 is able to modulate SMC phenotype, driving SMCs towards a less-differentiated and more proliferative phenotype and away from a more mature contractile phenotype [40]. To evaluate the effect of TWIST1 on SMC phenotype, we performed knockdown experiments in HCASMC and overexpression and knockdown experiments in rat aortic smooth muscle cells (A7r5). *TWIST1* knockdown using siRNA decreased proliferation in HCASMC as assessed with EDU assay (Fig 5C). In a cell counting assay, ectopic *Twist1* overexpression, which increased expression 5-fold, increased SMC number compared to control whereas *Twist1* siRNA knockdown decreased cell number over time (Fig 5D and 5E, S12 Fig). As SMCs become mature and differentiated, they can become less proliferative and transition towards a contractile and even osteoblast-like phenotype [41, 42]. We therefore hypothesized that *TWIST1*, in driving SMCs to a less differentiated and more proliferative state, would decrease the ability of A7r5 SMCs to form calcifications in response to phosphate-rich media. Indeed, *Twist1* overexpression led to a decreased number of calcifications, whereas siRNA knockdown of *Twist1* increased the calcification potential of A7r5 cells (Fig 5F).

### TWIST1 expression in atherosclerotic lesions in vivo

We next examined TWIST1 in vascular tissues *in vivo*. In human coronary arteries, TWIST1 protein could be detected in different compartments of the diseased vessel such as adventitia, media, and the neointima (S13 Fig). *TWIST1* mRNA was significantly upregulated in human carotid arteries displaying atherosclerotic lesions in the Swedish BiKE study (fold change = 2.616, p-value = 0.0008, n = 137, Fig 5G) [43]. Interestingly, TWIST1 expression was higher in lesions categorized as asymptomatic based on absence of signs of a transient ischemic attack prior to endarterectomy (fold change = -0.754, p = 0.0275, n = 127, Fig 5H, S14 Fig). Immunostaining for TWIST1 in normal carotid arteries and in areas of atherosclerosis show that low basal TWIST1 expression in medial SMCs was increased in carotid plaques (Fig 5I and 5J).

## Discussion

Previous whole-transcriptome analyses of human coronary arteries and their associations with vascular diseases have been limited to whole tissue eQTL analyses [12]. However, transcriptome profiling of individual cell types of the human coronary artery vessel wall has two major advantages. It links individual genes to a distinct cell type and controls for environmental conditions. Because of the important role of both HCASMC and HCAEC in CAD, and their related developmental origin, we have focused our studies on these two cell types.

Using ASE analysis in HCASMCs and HCAECs, we identified several genes for which expression levels were modulated by common, non-coding variation in these cells, including tropomyosin 1 (*TPM1*), endothelial lipase (*LIPG*), C-X-C motif chemokine ligand 5 (*CXCL5*) and 16 (*CXCL16*), activated leukocyte cell adhesion molecule (*ALCAM*) and transcription factor 21 (*TCF21*). We found that the majority of ASE genes were also eGenes in arterial tissues in the GTEx data set and were commonly shared between all three arterial tissues in GTEx (aorta, coronary artery, and tibial artery). Myosin heavy chain 13 (*MYH13*) was the only gene in our study with allele-specific expression in HCASMCs and eQTL association exclusively in coronary artery. As this tissue has the lowest sample number of all three tissues (n_Aorta_ = 267, n_Coronary_ = 152, n_Tibial_ = 388) this observation is likely based on a biological effect rather than lack of power for the other vascular tissues.

Colocalization analysis also identified several significant associations between GWAS signals for four different vascular traits and GTEx eGene signals in three arterial tissues. Many of these genes, such as *TCF21*, *ADAMTS7*, *LIPA*, *PHACTR1*, *IL6R*, and *LRP1*, are well-established risk genes. However, we found that genes identified in colocalization analysis overlapped no more than two vascular disease traits. There was only a modest overlap between ASE and colocalization genes, with *TCF21* as a well-established risk gene, and *MFGE8* and *UFL1* as novel, high-confidence candidates. Colocalization analysis also suggested *TWIST1* as a causal gene in the *HDAC9/TWIST1* association locus for CAD and stroke. Genome editing validated this finding. Thus, while constitutive loss of *HDAC9* has been shown to protect from atherosclerosis in a mouse model [44] and may be involved in regulating disease risk at this locus, our data link the rs2107595 haplotype with *TWIST1* expression, suggesting a role for *TWIST1* in human vascular disease.

The GWAS lead SNP rs2107595 serves as an eQTL for the *TWIST1* gene in human aorta (GTEx data v7), and disrupting or silencing the rs2107595 locus decreases *TWIST1* expression. TWIST1 is a bHLH transcription factor known for its role in epithelial-to-mesenchymal transition during mesenchymal development and in cancer progression [45, 46]. It is expressed in the cardiac neural crest [47] and proepicardial organ [48], which are sources of smooth muscle cell progenitors during coronary development. More specifically, TWIST1 promotes expression of the CAD-associated gene *TCF21* in the pro-epicardial organ [48], and is thus involved in the development of coronary artery smooth muscle cells. It is also likely involved in diseased vessel wall SMCs where *TCF21* upregulation modulates gene expression, SMC phenotype, and CAD risk [49]. Though we focus on the role of TWIST1 in SMCs for these reasons, TWIST1 has been implicated in shear stress induced endothelial dysfunction [50, 51] and plays an important role in endothelial biology. In particular, our data suggest that rs2107595 is an eQTL for *TWIST1* in ECs. The region around rs2107595 is also DNase-hypersensitive in several endothelial cell types suggesting it also functions as an enhancer in ECs. However, RBPJ binding of this region is higher in SMCs than in ECs (Fig 4C) suggesting rs2107595 may affect EC *TWIST1* expression through alternate mechanisms which warrant further investigation.

**Fig 6:**
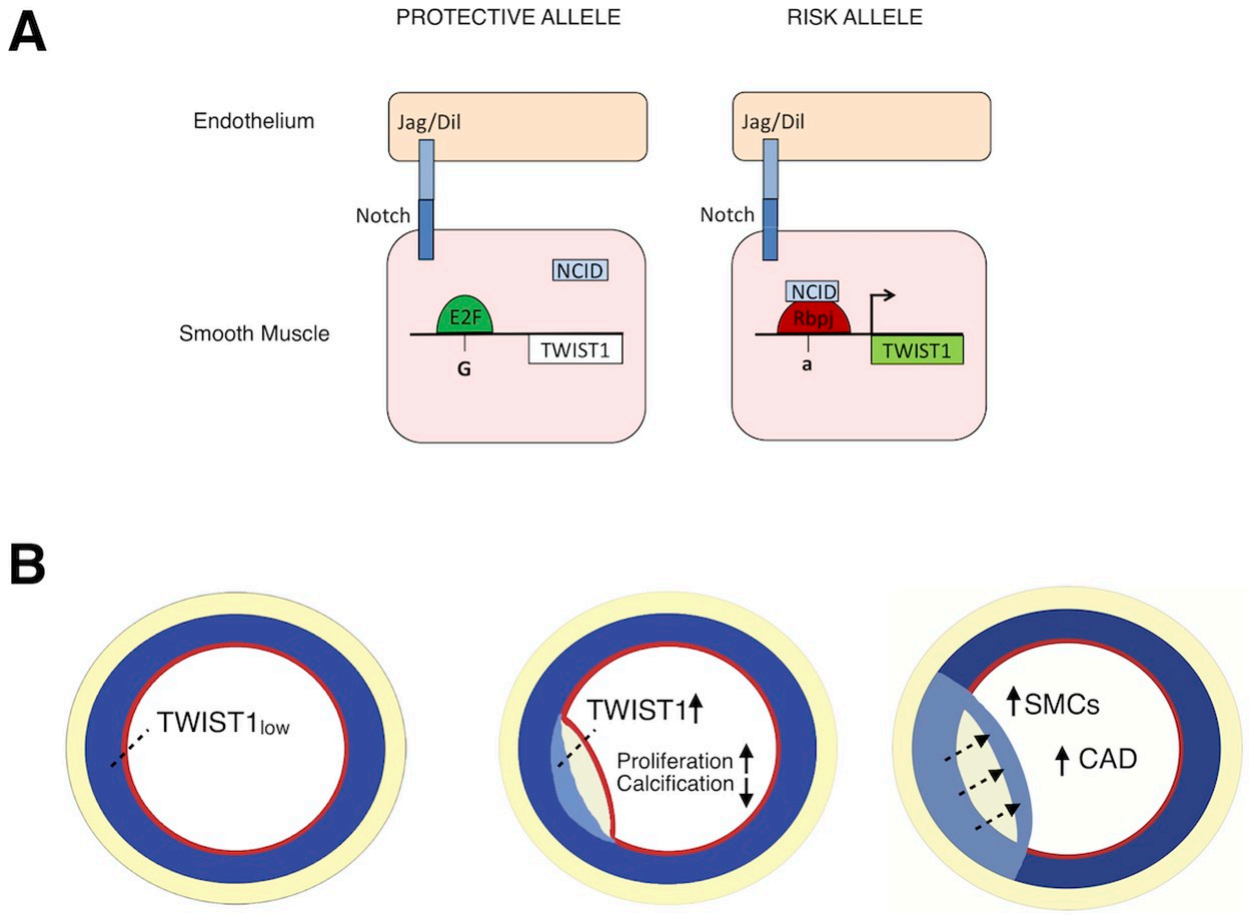
From SNP to phenotype. A) Proposed mechanism of transcriptional regulation at the rs2107595 locus in the vessel wall. The major allele (left) is predicted to bind E2F transcription factors which are unresponsive to Notch signaling. The minor allele (right) creates a consensus RBPJ binding site which promotes *TWIST1* gene transcription. B) The effect of TWIST1 protein on cardiovascular risk. In normal arteries, TWIST1 expression is low in fully differentiated smooth muscle cells of the tunica media (left). During early atherosclerosis (middle), there is increased TWIST1 expression in SMCs. This favors a de-differentiated SMC phenotype which increases SMC proliferation and increases coronary artery disease (right).

The rs2107595 risk allele is predicted to create an RBPJ binding site. RBPJ is a well-established transcriptional regulatory effector of the canonical Notch signaling pathway. Upon binding at consensus regulatory sites, it can serve as a transcriptional activator or repressor. We propose that upon Notch activation, the Notch intracellular cytoplasmic domain (NICD) can act as a coregulator for RBPJ, resulting in a switch from transcriptional repressor to activator (Fig 6A). Notch signaling has been shown to play a prominent role in smooth muscle biology, vascular development, and disease. Gene knockout experiments in mouse of *Notch* family members has shown severe vascular malformations, particularly affecting functionality of vascular smooth muscle cells [52]. For example *Notch3* is essential for the formation of functional arteries and mature smooth muscle cells [53], and *NOTCH3* mutations cause cerebral autosomal dominant arteriopathy (CADASIL) with severe alterations of small vessel smooth muscle cells in humans [54].

Notch signaling directly targets the *Twist1* gene via RBPJ binding at its promoter, leading to upregulation of *Twist1* expression in mouse limb bud mesenchymal progenitor cells (MPCs) [36]. Notch-mediated *Twist1* expression is necessary for repression of chondrogenic differentiation of MPCs. This described role for Twist1 in MPCs is in agreement with the expression profile of human *TWIST1* in our *in vitro* differentiation model. *TWIST1* expression is low in precursor cells and peaks at the neural crest progenitor stage. We see an upregulation of *TWIST1* expression in SMCs in response to IL-1β, which is in agreement with the model of smooth muscle de-differentiation and phenotypic switching during atherosclerosis [40]. In the context of human disease, we locate TWIST1 protein in the adventitia of human coronary arteries and in the tunica media and neointima of more complex atheromas. This is suggestive of a re-activation or upregulation of *TWIST1* in non-adventitial compartments and/or migration of *TWIST1* expressing cells into the atheroma. Similar to studies in developing heart valves [55] and cranial mesoderm [56], TWIST1 may activate downstream genes involved in proliferation, migration and extracellular matrix composition in the developing neointima of atherosclerotic lesions. This would promote SMC phenotypes such as proliferation and drive SMCs away from a differentiated phenotype capable of calcification. In support of this hypothesis we show that *Twist1* overexpression in rat aortic smooth muscle cells significantly increases cell proliferation and decreases calcification whereas *Twist1* knockdown by siRNA decreases proliferation and increases calcification.

The human genetic and genomic data in conjunction with *in vitro* studies reported here provide a compelling argument for the identification of *TWIST1* as a causal gene for vascular disease. The minor allele of the rs2107595 variant has been identified as a risk allele and is associated with increased *TWIST1* expression as shown by eQTL studies in vascular samples collected through GTEx and in cultured HCASMC. As expected, analysis of diseased human carotid artery samples revealed greater expression of *TWIST1*, compared to disease free vessels. However, *TWIST1* expression was found to be higher in carotid lesions in asymptomatic compared to symptomatic patients. Interestingly, this observation is consistent with the association of the disease allele with stable coronary artery disease over the infarction sub-phenotype [2]. The effects of modulating TWIST1 *in vivo* to potentially affect proliferation and calcification will likely yield new insights into the role of SMCs in atherosclerosis. While the number of HCASMC in plaque has been shown to be inversely correlated with disease risk, this association might break down in the case of unbridled proliferation of this cell type (Fig 6B). A salutary effect of HCASMCs in the plaque is hypothesized to result from the ability of medial SMCs to de-differentiate and migrate into the lesion where they subsequently re-differentiate and serve a stabilizing effect through cellular integrity and matrix production. Thus, *TWIST1* may play a more significant role in plaque burden than in plaque vulnerability. Additionally, though the role of calcification in plaque stability is complex and involves many cell types including SMCs [57], our data suggest *TWIST1* may affect the ability of SMCs to form calcifications.

In summary, we report unbiased transcriptional profiling of primary cells to identify novel risk genes for common vascular diseases with a focus on insufficiently researched, complex association loci. We propose *TWIST1* via the rs2107595 association as a novel risk gene for CAD, with a vascular causal mechanism related to SMC phenotype.

## Materials and methods

### Primary cell culture and sample processing

See supplemental materials for details (S1 File). In brief, primary human coronary artery smooth muscle cells (HCASMC) and human coronary artery endothelial muscle cells (HCAEC) were obtained from Lonza, Promocell, and Cell Applications (S1 Table). For RNA-Seq, Total RNA was isolated from confluent cells at passage 2 and prepared for sequencing using TruSeq Stranded Total RNA Library Prep Kit (Illumina RS-122-2103). The libraries were sequenced at the University of Pennsylvania Next-Generation Sequencing Core using paired-end, 100 and 125 nt long reads on an Illumina HiSeq 2500 system. Genomic DNA was isolated and genotyped on the Illumina Expanded Multi-Ethnic Genotyping Array (Illumina MEGA^EX^).

### Data analysis

Details of gene expression, gene ontology, pathway, sQTL, allelic-specific expression, and colocalization analyses are available in the supplemental materials (S1 File). The datasets generated during and/or analyzed during the current study are available in Gene Expression Omnibus under accession GSE111120 at https://www.ncbi.nlm.nih.gov/geo/query/acc.cgi?acc=GSE111120.

### Generation and analysis of CRIPSR lines

Genome editing of the enhancer region around rs2107595 was performed by CRISPR/Cas9 system as previously reported [58] using guide sequence GGATGAGGAGCCATTACTGT. In brief, HEK293 cells were seeded into 6 well plate (8×10^5^ cells /well) and cultured in DMEM with 10% FBS on day 0. On day 1, cells were transfected with 2 μg sgRNA/Cas9-GFP using 7.5 μl Lipofectamine 3000 per well. On day 2, cells were sorted using a Sony SH800s. A subset of the GFP-positive cells were singly sorted into a 96-well plate and expanded. Clones were screened for editing at rs2107595 as previously reported [59, 60] and outlined in S1. *TWIST1* and *HDAC9* gene expression was assessed in positively edited clones using qRT-PCR (S1 File), and aggregate data for clones with edited rs2107595 is presented.

### Biobank of Karolinska Endarterectomies (BiKE) study

Carotid endarterectomies (carotid plaques, CP) were collected at time of surgery from both symptomatic (S) and asymptomatic (AS) patients and retained within BiKE Study. One cohort (referred to as “large dataset”) consisted of Affymetrix microarray profiling of 127 atherosclerotic plaques (87 S and 40 AS patients) and 10 normal arteries. A second non-overlapping cohort (termed “small dataset”) included 50 plaques (41 from S and 9 from AS patients) and n = 5 normal arteries (S1 File).

### Ethics statement

Human studies from the Biobank of Karolinska Endarterectomies (BiKE) study are approved by the Ethical Committee of North Stockholm and performed with the following ethical permit numbers dating since 1995: BiKE EPN DNr 95–276/277; DNr 01–199; DNr 02–146; DNr 02–147; DNr 04–225/4; DNr 04–97 5T; DNr 2005/83-31; DNr 2007/281-31/4; DNr 2009/4:2; DNr 2009/9-31/4; DNr 2009/295–31/2; DNr 2009/512–31/2; DNr 2009/2000-32; DNr 2010/1022-31/1; DNr 2010/730-31/2; DNr 2011/196-31/1; DNr 2011/629–32; DNr 2011/950-32; DNR 2012/619–32; DNr 2012/916-31/4; DNr 2012/1096-31/2; DNr 2012/1279-32; 2013/615-31/4; DNr 2012/2188-31-5; DNr 2013/2048-32; DNr 2013/2137–32; 2015/1338-32; DNr 2015/2108-31/5; Dnr 2017/508-32. All human samples and data in BiKE are collected with written informed consent from patients or organ donors’ guardians. Tissue and blood sampling is conducted as part of the ordinary medical and surgical procedures and does not put the patients at unnecessary risk.

## Supporting information

S1 File Supplemental materials. Supplemental materials file includes additional detailed materials and methods.
(DOCX)

S2 File Differential gene expression. Differential Gene Expression file includes the list of genes that are differentially expressed between HCAECs and HCASMCs with |log2(fold change)|>2 and adjusted p-value < 0.001.
(XLSX)

S3 File Splicing quantitative trait loci analysis. The sQTL Analysis file includes the results from the sQTL analysis including cell-type and tissue-specific putative sQTLs passing FDR < 0.05 and Variant Effect Predictor.
(XLSX)

S1 Fig Gene expression from HCAECs and HCSMCs. A) Fold difference in expression of endothelial cell markers and smooth muscle cell markers from the RNASeq data in 19 paired samples. Endothelial genes are presented in black as the ratio of TPMs in HCAECs versus HCSMCs, and Smooth muscle genes are presented in grey as the ratio of TPMs in HCASMCs to HCAECs. B) Multi-dimensional scaling plot of the 500 most differentially expressed genes from GTEX RNA-Seq data from 205 aortic (pink) and 117 coronary artery (violet) samples as well as 19/20 in vitro cultured HCASMC (blue) and HCAEC (red) samples reveals significant overlap between human aortic and coronary artery tissues. HCAECs and HCSMCs cluster separately from each other with ECs showing tighter clustering among samples. Both types of primary cell lines cluster separately from arterial tissue, which may be due to the artificial nature of the *in vitro* environment.
(TIF)

S2 Fig sQTL analysis identifies loci associated with RNA splice variability. A) UpSet plot of genes with sQTLs in the HCSMC (SMC), HCAEC (EC) and GTEx datasets (FDR < 0.05, regression test). Vertical bars represent the count of unique genes per set. Below the bar graphs, each dot represents a dataset and intersecting sets are represented by lines connecting dots. Horizontal bars represent the total number of genes with putative sQTLs in each dataset. B) UpSet plot of all genes with putative sQTLs in the HCASMC/HCAEC and GTEx cohorts that colocalize with any signal for association with cardiovascular disease.
(TIF)

S3 Fig sQTL association between STAT6 and rs167769 in HCAEC and GTEx tibial artery. A) Splicegraph structure of STAT6 near the 5’ end, showing the implicated LSV targeting exon 6. Inset zooms in on the relevant exons and splice junctions (not to scale). B)—C) Scatterbox plots of PSI for the alternative 5’ splice site event in STAT6 exon 3, which is the first exon in the majority of transcripts of STAT6, using data from HCAEC and GTEx, respectively. Each plot represents samples of the indicated genotype at rs167769. Each green point represent inclusion level (PSI) quantified in a sample of the LSV’s green junction in A. D) RNA-seq reads mapping to the alternative 5’ splice site event at the canonical first exon of STAT6 (purple and green junctions in A). Tracks are labeled with the dataset of origin and sample genotype at rs167769 (HCAEC) or rs324011 (GTEx coronary artery). Representative samples were randomly selected from the pool of all samples with the indicated genotype in their respective dataset. Reads mapping into the canonical exon body are outlined in a red box. Reads mapping to the 18-nt extension are immediately to the right of this box. The UCSC transcript annotation track is depicted on the bottom for reference; the bottommost transcript uses a different first exon not depicted.
(TIF)

S4 Fig ASE/Colocalization association plots for UFL1, MFGE8 and TCF21 loci, red dot represent p value < 5e-08 and blue dots represent p value < 1e-06. (TIF)

S5 Fig Expression of TWIST1 in HCASMCs and human carotid plaque samples. A) TWIST1 is increased in SMCs relative ECs based on both RNASeq data and qRTPCR. Immunocytochemistry shows nuclear TWIST1 staining in ACTA2-positive SMCs. B) Expression of TWIST1 is positively correlated with SMC markers (red) and negatively correlated with EC and immune cell markers (blue) in human atherosclerotic plaque samples from the BiKE study.
(TIF)

S6 Fig Genomic neighborhood and PheWAS of rs2107595. A) UCSC Browser view displaying the genomic landscape around GWAS SNP rs2107595 (box). H3K27ac histone modification ChIP-Seq data from bone-marrow derived mesenchymal stem cells from the ENCODE project is also displayed. There is high H3K27ac at this locus which indicates that this area is likely an active enhancer. B) Phenome-Wide Association Study data from the UK Biobank shows that rs2107595 is significantly associated with three common vascular disease phenotypes.
(TIF)

S7 Fig Overview of CRISPR/Cas9 genome editing near rs2107595 and the effect of disrupting rs2107595 on expression of *TWIST1* and *HDAC9*. A) The wildtype sequence shows rs2107595 (yellow), PAM sequence (red) and the cut site within the guide RNA sequence (blue arrowhead). Representative sequences for editted clones are aligned below to show the effects of CRISPR/Cas9 editing in this genomic region. B) A summary of all clones generated and used for analysis show a range of insertion/deletions. In all cases, rs2107595 is either missing or editted (as in clone F2). C) *TWIST1* and *HDAC9* gene expression is shown for each cell line relative to control. Disruption of rs2107595 decreased *TWIST1* expression in most cell lines.
(TIF)

S8 Fig *In Silico* analysis of transcription factor binding at rs2107595. A) Overview of proposed transcription factor binding to the major and minor alleles based on Transfact Professional (2014.4 data release). B) Using JASPAR, an open-access database of transcription factor binding profiles, we find that if there is a G at the rs2107595 locus (and C on the complementary strand), this base pair forms part of an E2F binding motif. The E2F position matrix shows that if this is converted to a T, E2F will bind ~ 5% of the time (top yellow box). When the minor allele is present, and there is a T on the complementary strand of the locus, this base pair forms part of an RBPJ binding motif. The corresponding RBPJ position weight matrix shows that a C at this position would result in RBPJ binding ~ 0.5% of the time (bottom yellow box).
(TIF)

S9 Fig Chromatin-Immunoprecipitation (CHIP) qPCR analysis of RBPJ and E2F binding. A) ChIP was performed using RBPJ antibody and IgG controls on heterozygous HCASMCs and HCAECs. A region 17kb upstream of rs2107595 was amplified to serve as negative control. As expected, there was no significant enrichment of this area with RBPJ relative to IgG control. B) ChIP was performed using three different E2F antibodies (E2F1, E2F2, E2F4) as well as IgG control on heterozygous HCSMCs. qPCR of the region around rs2107595 shows significant enrichment with E2F4 suggesting E2F4, but not E2F1 or E2F2, binds this region.
(TIF)

S10 Fig ChIP-PCR restriction fragment length polymorphism analysis. Chromatin-Immunoprecipitation was performed using RBPJ on heterozygous HCASMCs. The region surrounding rs2107595 was amplified and cloned into sequencing vectors. PCR digestion of 22 colonies show that RBPJ is preferentially binding the risk (A) allele (17/22 colonies).
(TIF)

S11 Fig MYH11 expression in neural crest progenitor cells and derived smooth muscle cells with or without IL-1β treatment. Progenitor cells express less MYH11 than derived SMC cells, and that MYH11 does not increase in response to IL1B.
(TIF)

S12 Fig Ectopic TWIST1 overexpression and siRNA knockdown. A) CMV6-promoter driven TWIST1 overexpression ((pTWIST1) resulted in a ~ 5-fold increase in TWIST1 gene expression. B) TWIST1 protein is increased as well in HEK293T cells. WM: Western Marker, PM: Prestained Marker. C) SiRNA knockdown of TWIST1 resulted in ~95% decrease in TWIST1 gene expression. D) TWIST1 overexpression and knockdown at 24H and 48H had no consistent effect on apoptosis or necrosis.
(TIF)

S13 Fig TWIST1 expression in atherosclerotic human coronary arteries samples. TWIST1 protein (blue) is present in the media (M) and in the plaque. L = Lumen; N = Neointima, M = Media; A = Adventitia.
(TIF)

S14 Fig TWIST1 expression in human endarterectomy samples, small cohort replication. A) Twist1 is higher in carotid plaques relative to normal arteries. B) There is a trend towards decreased Twist1 expression in symptomatic lesions relative to asymptomatic lesions.
(TIF)

S1 Table Primary human cell lines. (XLSX)

S2 Table Pathway analysis of genes overexpressed in HCAECs. (XLSX)

S3 Table Summary of sQTL results that colocalize with GWAS loci for vascular disease for both primary culture data form HCSMACs and HCAECs and for GTEx arterial tissue (CA = coronary artery, TA = tibial artery). Each row represents an association statistic between a SNP and a local splicing variation (LSV) within a gene. SNP-gene pairs are filtered such that if a SNP is found to associate with multiple LSVs in the same gene, only the most significant association is reported. It is expected that LSVs within the same gene may highly correlated. The full sQTL output for all tests is attached as supplemental file (S3 File) and includes all SNP-LSV pairs passing the indicated significance threshold even in cases where the SNP associates with multiple LSVs in the same gene.
(XLSX)

S4 Table ASE in heterozygous HCASMC and HCAEC lines. Table includes the position of all SNPs that showed ASE, corresponding gene annotation, cell type in which ASE was observed, whether this was a GTEx eGENE or GWAS colocalization site, and the average number of reads obtained for the major or reference (REF) and the minor or alternate (ALT) alleles. GTEx eGene tissues: A = Artery_Aorta; C = Artery_Coronary; T = Artery_Tibial. GWAS colocalization traits: CAD = Coronary Artery Disease; MIG = Migraine.
(XLSX)

S5 Table Colocalization analysis of 4 vascular GWAS trait associations and 3 arterial GTEx eQTL signals. (XLSX)

S6 Table Significant colocalization of top GWAS SNPs with GTEx eQTLs. (XLSX)
