# Supplementary material for: Genomic profiling of human vascular cells identifies TWIST1 as a causal gene for common vascular diseases
Source: PLoS Genet. 2020 Jan 9;16(1):e1008538. doi: 10.1371/journal.pgen.1008538 (PMC6975560; doi:10.1371/journal.pgen.1008538)
Supplement: S1 File — Supplemental materials file includes additional detailed materials and methods. (DOCX) [file pgen.1008538.s001.docx]

**Supplemental Materials**

**Materials and Methods**

*Cell culture*

Donor-matched pairs of primary human coronary artery smooth muscle cells (HCASMC) and primary human coronary artery endothelial cells (HCAEC) were obtained from Lonza, Promocell, and Cell Applications (Supplemental Table 1). HCSMCs were cultured in Clonetic SmGM-2 Growth Medium (Lonza CC-3182). HCAECs were cultured in Clonetic EGM-2 MV Growth Medium (Lonza CC-3202). HEK-293T and A7r5 cells were maintained in DMEM, high glucose (Thermo Fisher Scientific 11965084) plus 10% FBS (HyClone SH30910.03 AAD202492). No antibiotics were used in any culture. Primary HCAECs were cultured, processed and sequenced at Penn. Primary HCASMCs were cultured at Stanford. Cell lysates were shipped to Penn and further processed in parallel with primary HCAECs. Primary HCAECs were used up to passage 5 from the original vial; for next-generation sequencing purposes cells were harvested at confluency at passage 2.

*RNA and gDNA isolation*

Total RNA for qPCR was isolated using the Quick-RNA MicroPrep kit (Zymo Research R1050) according to the manufacturer’s protocol. Large RNA (>200nt) for RNA-Seq was isolated with the NucleoSpin miRNA kit (Macherey-Nagel 740971.50). All isolated RNA samples had an RNA Integrity Number (RIN) of 10 on the Agilent Total RNA Nano chip and 28S/18S ratios were not significantly different between cell types. Genomic DNA for genotyping was isolated using the Quick-gDNA MiniPrep kit (Zymo Research D3024).

*Genotyping*

DNA samples from eighteen subjects (15 men, 3 women) were genotyped on the Illumina Expanded Multi-Ethnic Genotyping Array (Illumina MEGA^EX^). The samples were processed using the Illumina released beadpool manifest and clustered using a custom cluster file generated by the Center for Applied Genomics (CAG) at the Children’s Hospital of Philadelphia based off of approximately 400 samples processed on the same array. The genotypes were parsed from GTC files generated during the scanning process in accordance to the Illumina LIMs software. The released Plink files were gathered and processed using CAG Perl scripts. After which Plink software was used to generate the bfiles. The final Forward allele bim file was converted using CAG Perl scripts and the Illumina provided strand orientation data. Standard GWAS quality control measures were applied to the data [1]. Subjects were screened for discordant sex, excessive heterozygosity (greater than 3 standard deviations from the mean), genotype missingness (> 5%), and for relatedness. No subjects were removed for these criteria.

At the SNP level, 1,041,334 SNPs were unobserved in the data set, and autosomal and duplicate SNPs were removed as well (29,839 and 11,262 SNPs, respectively). SNPs were additionally screened for minor allele frequency (<0.01; 392,524 SNPs removed). After quality control, the remaining 560,504 SNPs were imputed against the 1000 Genomes Phase III reference panel [2] using the Sanger Imputation Service Data [3] was phased using EAGLE2 [4], and imputed using PBWT [5]. 81,706,022 imputed SNPs underwent further quality controls measures, including removal of SNPs with INOF score<0.4, multi-allelic SNPs and non-SNP variants as well as SNPs with fewer than two minor allele copies in the data set. A total of 15 subjects and 9,629,888 SNPs passed QC and were used in the final analysis.

*RNA-Sequencing*

All samples were sequenced at the Next-Generation Sequencing Core at the University of Pennsylvania Perelman School of Medicine. RNA-Sequencing was performed as paired-end, 100 and 125 nt long reads on an Illumina HiSeq 2500 system from libraries generated from 300 ng of RNA using the TruSeq Stranded Total RNA Library Prep Kit (Illumina RS-122-2103) according to the manufacturer’s protocol.

| **HCAEC** | **mapped fragments** | **HCASMC** | **mapped fragments** |
| --- | --- | --- | --- |
| 317988 | 68368809 | 317155 | 94148815 |
| 3101801 | 63256864 | 3101801 | 83099207 |
| 1522 | 54331924 | 1522 | 76718152 |
| 1596 | 65477974 | 1596 | 67104517 |
| 1848 | 68966041 | 1848 | 60136236 |
| 1858 | 71284623 | 1858 | 149385449 |
| 2102 | 76634718 | 2102 | 58103662 |
| 2105 | 65304846 | 2105 | 91358750 |
| 2139 | 82051204 | 2139 | 55688144 |
| 2161 | 73359223 | 2161 | 103275605 |
| 2228 | 58948900 | 2228 | 58476869 |
| 2305 | 77011369 | 2305 | 58844375 |
| 2315 | 65378302 | 2315 | 83119454 |
| 2435 | 56974735 | 2435 | 70630303 |
| 2463 | 67711948 | 2463 | 69037126 |
| 2989 | 71350494 | 2989 | 68876218 |
| 2999 | 70957950 | 2999 | 66290555 |
| 3003 | 62498623 | 3003 | 51633229 |
| 1060602 | 88903842 | 1060602 | 54453975 |

*Gene expression and pathway analysis*

The MDSplot of leading transcript differences between HCASMCs, HCAECs and GTEx tissues (liver, whole blood, coronary artery, aorta) was generated using edgeR v3.8.6 (R-3.1.2) from transcript abundances from fastq files using kallisto v0.42.1 (kallisto quanti -i -o -b 100). Tagwise and common dispersion was calculated using the GLM method.

For differential gene expression, principle component, gene set enrichment and pathway analysis fastq files were aligned to GRCH37.p13 using STAR v2.3.0d (--outFilterMultimapNmax 10 --outFilterMismatchNmax 10 --outFilterMismatchNoverLmax 0.3 --alignIntronMin 21 --alignIntronMax 0 --alignMatesGapMax 0 --alignSJoverhangMin 5 --alignSJDBoverhangMin 3) and converted using bamtools v2.3.0.

Differential gene expression and principle component analysis was performed with easyRNASeq v2.8.2 and DESeq2 v1.15.28 (R-3.3.0), and p-values were adjusted using Bonferroni correction to generate list of genes differentially expressed between HCASMCs and HCAECs. Pathway analysis was performed using Ingenuity IPA (Fall Release September 2016).

*Gene set enrichment analysis*

The top 500 of 1982 genes preferentially expressed in HCASMC (also expressed in coronary artery and aorta) or 966 genes preferentially expressed in HCAEC (also expressed in coronary artery) were tested for enrichment in GSEA hallmark gene sets (GSEA software v2.2.23) using a gene list pre-ranked on log2 expression fold changes between HCASMCs and HCAECs from the differential gene expression analysis.

*Splicing Quantitative Trait Locus (sQTL) analysis*

We ran MAJIQ 1.1.3a builder on RNA-seq data from 19 subject-matched human coronary artery (HCA) smooth muscle cells (SMC) and epithelial cells (EC). The builder created a database of local splicing variations (LSVs), or percent spliced in (PSI) of alternatively-spliced mRNA segments, which were then quantified and tested for sQTL. The LSV database was based on an ENSEMBL gff3 annotation for GRCh37 (hg19) combined with junction spanning reads from the RNA-seq samples. For further analyses, we only included 14 subjects with genotypes available for sQTL mapping. Next, we used MAJIQ to estimate the percent splice inclusion (PSI) levels for each splice junction in each LSV in each sample, using default parameters [6]. Then, we collected the expected PSI values per junction from all samples for which the LSV is quantifiable according to MAJIQ’s default settings. Next, we scanned for genomic variants (SNPs) within a contiguous region containing all exons involved in the LSV plus 1 kB upstream and downstream. We discard any SNP that does not have at least three subjects with heterozygous genotypes and at least three subjects with homozygous minor alleles before imputation. To mitigate computational burden and the high correlation between PSI values of junctions of the same LSVs only (perfect correlation for binary events by construction), we kept the junction with the greatest variance among per LSV by PSI expectations across samples. For the sQTL mapping, we transform PSI values with an inverse sigmoid (logit) function, perform quantile normalization to N(0, 1), and regress out a matrix of fixed covariates learned from genotype-based principal components to account for potential population substructure [7]. Finally, we compute the test statistic and associated p-value for linear association between transformed PSI and imputed genotype (see Supplemental material for additional analysis using a one-way F-test for which subjects missing genotype are discarded). We then applied multiple testing correction by gene and considered FDR < 0.05 as genome-wide significant sQTLs. We used all nominally significant sQTL (unadjusted p < 0.05) for replication analyses using GTEx artery tissues. Untrimmed GTEx samples were mapped to GRCh37 using STAR v2.3.1a --alignSJoverhangMin 8 --outSAMtype bam unsorted and sorted using samtools sort.

We collected seven GWAS summary statistics from meta-analyses of cardiovascular diseases [8-10]. From each study, SNPs reaching genome-wide significance (p < 5 x 10^-8^) were selected, in addition to all variants in linkage disequilibrium (LD) with those SNPs (r^2 ≥ 0.8). The sQTL pipeline described above was executed with the SNP search space restricted to only those variants in LD with GWAS SNPs for each disease separately. Given the high correlation by construction between SNPs, junctions of each LSV and between LSVs in the same gene, only pairs where the gene-based corrected FDR < 0.05 were reported. We also applied the same constraints as in the whole-genome sQTL scan, where a SNP and LSV need to be within 1 kB of each other and SNPs were filtered for variants with at least three subjects in each of three genotypes: reference-reference homozygote, heterozygote, and alternate-alternate homozygote. As a result, two out of seven GWAS (migraine and coronary artery disease) remained for further analyses.

We filtered the same ENSEMBL gff3 annotation to include only the genes identified in the SMC/EC scans as harboring nominally-significant (p < 0.05) sQTLs. This annotation was then used with MAJIQ 1.1.3a to analyze 745 GTEx samples representing three arterial tissues: aorta (N=247), coronary (N=141), and tibial (N=357). Finally, the above procedure for calling sQTLs was performed for this GTEx build.

The Python package scipy-1.1.0 was used for the statistical tests, statsmodels-0.9.0 was used for the FDR correction, scikit-learn-0.19.2 was used for genotype imputation, and UpSetPlot-0.1 to plot intersecting sets and counts of sQTLs and sGenes across different cell types.

Additionally, we tested 26769 SNPs which were identified as putative sQTLs in GTEx for predicted splicing effect using the ENSEMBL Variant Effect Predictor (VEP) on GRCh37. Default parameters were used to run VEP via the web interface, with MaxEntScan analysis enabled. MaxEntScan scores a splice site “strength” using a fixed kmer window around the 5’ or 3’ splice site. VEP considers all SNPs within the appropriate kmer window (5’SS: last 3 bases of exon → first 6 bases of intron; 3’SS: last 20 bases of intron → first 3 bases of exon) for annotated exon boundaries. In all, 488 SNPs in 400 genes passed this filtering criteria. The reported score is the change in entropy resulting from changing the reference allele at each variant to its recorded alternative allele. A positive score indicates that the reference allele reduces splice site utilization over the alternative allele at that site.

*Allele-Specific Expression Analysis*

ASE analysis was performed using the beta-binomial model implemented in QuASAR.[11] In brief, for each sample, allele-specific RNA-seq read counts were generated at all heterozygous variants with SAMtools.[12] Only uniquely mapping reads with a base quality ≥10 at the variant were counted, and only those variants with coverage of at least eight reads were reported. False discovery rates were estimated using the method of Storey [13]. Comparison of genotypes derived from RNA-Seq data and SNP chip based imputation demonstrated an absence of sample swaps.

*Colocalization Analysis*

The Genotype-Tissue Expression (GTex) project is a large effort to genotype and corresponding gene expression data across tissues and individuals which has been expanded to include dozens of tissue types from hundreds of human tissue donors. In order to discover relationships between common vascular traits associated SNPs and eQTL findings in GTEx version 7 tissues [14], we performed co-localization analysis using the approximate bayes factors method as implemented in the coloc.abf function in coloc package[15] in R version 3.2.2.

SNPs associated with vascular traits were obtained from 4 published genome-wide association studies: Abdominal Aortic Aneurysm, Coronary artery disease, Migraine and Stroke. At each genome-wide significant locus, a single lead SNP was selected. We assessed evidence of co-localization by using all SNPs that were within ±1 Mb of the lead GWAS variant and were in common between the GWAS and GTEx eQTL study.

Summary statistics from genome-wide association studies for CAD [16], Stroke [10] and Migraine[8] were obtained online:

| Trait | Website | File(s) |
| --- | --- | --- |
| CAD | http://www.cardiogramplusc4d.org/data-downloads/ | cad.additive.Oct2015.pup.zip |
| STR | http://cerebrovascularportal.org/informational/downloads | 3490334.Traylor.2012.zip |
| MIG | http://www.headachegenetics.org/content/datasets-and-cohorts | any_mig.gwama_.out_.isq75.nstud12.clean_.p1e-5_3.txt |

Summary statistics for Abdominal Aortic Aneurysm [17] were provided by Phillip Haycock (University of Bristol, UK, file: AAA_MetaGWAS.gz).

*Conditional Analysis*

Utilizing LD information estimated from a reference panel and summary-level statistics which comprise effect size, standard error, p-value and effective sample size, GCTA [18] allowed us to perform conditional analyses at the HDAC9-TWIST1 locus. To produce a reference panel that mimics the structure of the effective sample sizes of the CARDIoGRAMplusC4D 1kg effort, we sampled with replacement 3511 EUR, 96 AMR, 360 EAS, 984 SAS and 50 AFR individuals from the 1000Genome Phase3 v5 ethnic-specific reference panel. To identify the potential secondary signal at this locus, the associations with CAD of the SNPs in the region was evaluated when they were conditioned upon the most significant SNP at this locus.

*Data Availability*

The datasets generated during and/or analysed during the current study are available in Gene Expression Omnibus under accession GSE111120.

*Quantitative Realtime PCR (qRT-PCR)*

QRT-PCR was performed using either Taqman reagents and probes or SYBR green reagents and primers, as outlined below. For Taqman, Reverse-Transcription of RNA samples was carried out using the SuperScript III First-Strand Synthesis System (Thermo Fisher Scientific 18080051) according to the manufacturer’s protocol. A standard dilution series of cDNA generated from XpressRef Universal Total RNA (Qiagen 338112, 338116) was used for QRT-PCR. Genes of interest plus 3 reference genes (Actb, Gapdh, Tbp) were measured using Taqman probes and TaqMan Fast Advanced Master Mix (Thermo Fisher Scientific 4444965) in a QuantStudio 7 Flex Real-Time PCR System (Thermo Fisher Scientific 4485701).

| Target gene | Species | Taqman probe ID |
| --- | --- | --- |
| ACTB | Human | Hs01060665_g1 |
| GAPDH | Human | Hs02758991_g1 |
| TBP | Human | Hs00427620_m1 |
| TWIST1 | Human | Hs01675818_s1 |
| HDAC9 | Human | Hs00206843_m1 |
| actb | Rat | Rn00667869_m1 |
| gapdh | Rat | Rn01775763_g1 |
| tbp | Rat | Rn01455646_m1 |
| twist1 | Rat | Rn00585470_s1 |
| hdac9 | Rat | Rn01499096_m1 |

Relative quantities were calculated by dividing the measured quantities of target genes by the combined average quantities of the three reference genes. Errors were calculated as root-mean-squared from the triplicate measurements of all four genes (target gene plus 3 reference genes).

For SYBR experiments, total RNA was extracted using the RNeasy mini kit (Qiagen 74104). cDNA was synthesized from 250 ng total RNA using the Maxima First Strand cDNA Synthesis kit (Thermo Fisher Scientific K1671). qRT-PCR reaction mixtures were prepared with SYBR green PCR master mix (Applied Biosystems) and run on the 7500 Fast Real-time PCR system. C_T_ values were normalized to porphobilinogen deaminase (PBGD). Primer sequences are listed below and the corresponding melt-curves are shown below:


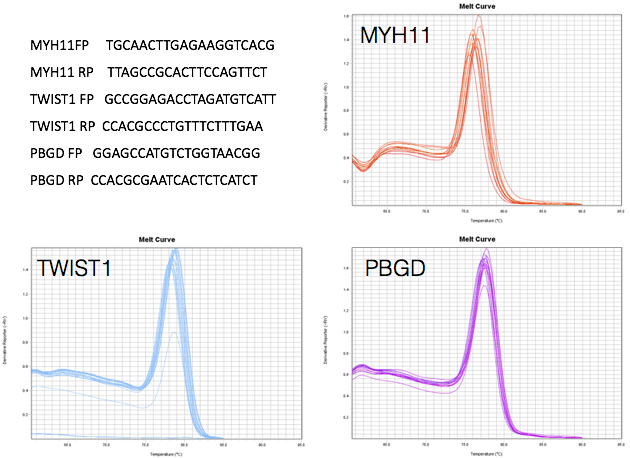


*Dual luciferase reporter assay*

A 800 bp long genomic region centered on the association SNP rs2107595 was cloned into pLuc-MCS (Agilent 219087). The SNP genotype was altered with the QuikChange II XL Site-Directed Mutagenesis Kit (Agilent 200521) and the altered region subcloned back into the empty pLuc-MCS backbone to ensure no additional changes had been introduced the vector backbone which might alter its transcriptional activity. 400,000 cells were then transfected with 200 ng pLuc-MCS and 10 ng pGl4.74 (Promega E6921) using Lipofectamine 3000 (Thermo Fisher Scientific L3000150). Cells were harvested 24 hours post transfection and relative luciferase activity measured in a BioTek Synergy Mx microplate reader using the Dual-Luciferase Reporter Assay System (Promega E1960). pGl3-Control (Promega E1741) was used as a positive control vector for strong enhancer activity.

Dual luciferase reporter region: GCTAGTCATTCCATTACTTGAAAGAGCTCCTCATTGTGGATAATGGGCAGCCAGTGCCTTAACAGTTTTTGACAAACTGAAAATGATTAAAAAGTTTGCAGGCTGCTTCCGGAGAGAAATTACAAATTAGAAACACATAGATCATCTGCAGAGATTTGATGTTTAATGCACCCACTGAAAACTGTCTCTGACAATGGGTGCTGGTAGTCCTTTCTTTGGTGGTATGTGTTTAATCTTATTGTTCGCATCAAATAAAAAGAGAAGGGAATAGTAGTGAGTGCCACTTGTCTGAACCTTAGAAATGAGGTTCATTTTCTTCACTGTCAAAACATGACAGGAAAACAGATACTCGTTACATGTTGAAGAGTGAAAGATTTGAGCTAATGCTTGGCAGGAACATACATAGCTGAGGTTTCATTTCTTTGTACGTACTGTGGCCATGTTAAAGTTCAGTCTTCACATTCCTTTCTGGTGAAAAAGAGATTTAATGAACATGCTACATGTTTCTTTTAGCTGCTAAATGACATGCGTCTTCTCCTTATGACTACTTTTTTTTGAGTATCTTGGAAATATTGTGTGTGGCTATCTTTCTGACTAAAAAGTACTCATTGAGAAGGATGAGGAGCCATTACTGTGGGACAAAAACATTTTCCC**R**CAAAAAAGAATGTACAAGCACACAAAAAAGATGTGGGATTTTTATGAATCATATCCTGTAATTTTTCACTTTTTTTCCAGCTACCTCCTTCTCAGAACAGAATCCAAGAAAACCAAAGTAAACTATTTGTGCTGTGTGCAAAAATTTTGCCAA

Transactivation assays were carried out by co-transfecting 500 ng of the constitutively active Rbpj expression construct pCMX-RBPJ-VP16[19] or pCMV6 empty vector (OriGene PS100001). As a positive control for transactivation by Rbpj a plasmid containing the luciferase gene driven by a beta globin minimal promoter as well as a hexamer of the two RBPJ binding sites of the TP1 promoter [20]. Experiments were carried out in triplicate and in least three independent experiments.

*Chromatin immunoprecipiation*

ChIP was carried out as previously described [21], using a rabbit polyclonal antibody raised against RBPJ (Abcam ab25949, lot number GR267193-1). Cell lines that were heterozygous for the rs2107595 locus were used.

*ChIP-qPCR and allele-specific ChIP-PCR-RFLP*

Enrichment of the association SNP containing region was assessed by SYBR-qPCR using the iTaq Universal SYBR Green Supermix (Bio-Rad 172-5121). The target region was amplified using TGACTAAAAAGTACTCATTGAGAAGG and GGATATGATTCATAAAAATCCCACATC. A 17 kb upstream negative control region was amplified with GATATTCCTGGCTTCGTATAATGG and TCTAAGGTTTCATTGTCTGACCTAG. A dilution series of sheared chromatin (Input) was used to adjust for differences in amplification efficiencies.

To assess allele-specific Rbpj binding, the region harboring rs2107595 was amplified from the ChIP samples with GCAGGAACATACATAGCTGAGG and CATGCCTAAGTACACACACTGTAC, A-tailed and subcloned into pGem-T Easy (Promega A1360). After colony PCR, PCR products were purified (Zymo Research D4004) and digested with FauI (NEB R0651S) which cleaves the PCR product only in presence of the rs2107595 major (G) allele. Results of digested samples and controls were visualized on agarose gel.

*CRISPR-Cas9 Genome editing and CRISPR inhibition of HCASMC*

Clonal cell lines with CRISPR/Cas9-mediated genome editing near rs2107595 were generated as follows. Following transfection with CRISPR-Cas9 system, a subset of the GFP-positive cells were singly sorted into a 96-well plate and expanded until clones reached about 70% confluence (~ 2 weeks). Cells were then split 1:2 into two fresh 96-well plates. Once the majority of the wells obtained 80% confluence, one plate was frozen and the other was used for genomic DNA extraction, as previously reported [22, 23]. Briefly, cells were washed with 100ul / well PBS, 50ul Lysis buffer (10mM Tris pH7.5, 10mM EDTA pH8.0, 10mM NaCl, 0.5% N-lauroylsarcosine, and 1 mg/ml Proteinase K was added to each well. Following overnight incubation at 55°C, 100ul of ice cold 75 mM NaCl in ethanol was added and mixed well. Plates were then incubated at room temperature for 2 hours, and the solution was discarded by inverting plates. Wells were washed with 100ul 70% ethanol twice. Plates were allowed to air dry for 30 minutes and then precipitated DNA was dissolved in 30-50ul TE. The targeted region was amplified using 10ul gDNA, forward primer GCTTGGCAGGAACATACATAGC, and reverse primer AGCCAAGTTTCATGCCTAAGT and Q5 Taq DNA polymerase (NEB) as per manufacturer’s instructions. Cycling conditions were as follows: 98°C for 30s, 30 cycles of 98°C for 5s, 65°C for 30s, 72°C for 30s, and one cycle of 72°C for 2 min. All wells showing a positive band at about 600bp were Sanger sequenced with forward primer. Positively edited clones were then thawed and expanded for total RNA extraction using RNeasy micro kit (Qiagen, #74004) according to manufacturer’s instructions. *TWIST1* and *HDAC9* gene expression was assessed using qRT-PCR as outlined above, and a mean value for clones with edited rs2107595 is reported and presented in graphical format.

Epigenetic editing of HCASMCs was performed with dCas9-KRAB [22]. The guide for rs2107595 (GGATGAGGAGCCATTACTGT ) was cloned into Addgene plasmid #71237 using BsmBI sites. Second generation lentivirus packaging vectors were used to transfect HEKs using Lipofectamine 3000, as per manufacturer’s instructions. Fresh media containing 1/500 ViralBoost Reagent (Alstem, catalog # VB100) was added to the cells twenty-four hours post transfection. Viral supernatant was collected, filtered and frozen three days after transfection. HCASMCs were seeded at 5x10^4^ cells per well in a 6-well plate. Media was removed and replaced by viral supernatant supplemented with 8ug/ml polybrene the following day. Viral supernatant was removed and replaced with fresh maintenance media after twenty-four hours. Three days post transduction cells were harvested for RNA extraction using Qiagen MicroRNA kit (Catalog # 74034). cDNA was synthesized using Thermo Fisher Scientific High Capacity RNA-to-cDNA kit (Catalog # 4387406) with 250ng RNA. Twist1 expression was measured using Thermo Fisher Scientific Taqman Probe Hs00361186_m1.

*Proliferation Assay*

A7r5 cells were transfected in 10cm dishes at 90% confluency with 20ug pCMV6-TWIST1 (Origene RC202920) or pCMV6-empty for 4 hours using Lipofectamine 3000 (Thermo Fisher Scientific L3000015). After 1 hour of recovery, cells were detached, counted and 10,000 cells seeded into 6-well plates. Cells were harvested and counted 24h, 48h, 72h and 96h after start of transfection. For siRNA knockdown, A7r5 cells were transfected in 12-well plates at 70% confluency with TWIST 1 siRNA targeting the coding sequence of Twist 1 (Integrated DNA Technologist rn.Ri.Twist1.13.2) or scrambled control (Integrated DNA Technologies NC-1) using Lipofectamine RNAiMax (Life Technologies 13778075) for 24 hours. Cells were detached and counted at 24h, 48h, 72h, and 96 h after start of transfection. Experiments were carried out in triplicate and as 3 independent experiments. Necrosis and apoptosis was assessed using Annexin V and Propidium Iodide staining (Sigma-Aldrich #APOAF-20TST) and quantified on BD Accuri C6 Flow Cytometer.

*HCASMC EDU Assay*

Human coronary artery smooth muscle cells were seeded at 8 x 10^5 cells per 6 well. Twenty four hours later cells were transfected with siRNA against TWIST1, purchased from Origene catalog # SR304977, using Thermo Fisher Lipofectamine RNAiMAX reagent, as per manufacturer instructions. Cells were allowed to incubate with transfection reagents overnight, after which media was changed. Twenty four hours after media change, EdU assay was performed with Thermo Fisher Click-iT Alexa Fluor 488 Imaging Kit, catalog # C10337 as per instructions. Cells were mounted with Vector Laboratories Vectashield Mounting Medium with DAPI, catalog # H-1200. Images were captured on a Leica inverted microscope. Images were analyzed and nuclei counted using ImageJ.

*Calcification Assay*

A7r5 cells were transfected in 6-well plates with 1ug of pCMV6-TWIST1 (Origene RC202920) or pCMV6-empty. For siRNA knockdown, A7r5 cells were transfected in 12-well plates at 70% confluency with TWIST 1 siRNA targeting the coding sequence of Twist 1 (Integrated DNA Technologist rn.Ri.Twist1.13.2) or scrambled control (Integrated DNA Technologies NC-1) using Lipofectamine RNAiMax (Life Technologies 13778075) for 24 hours. Following transfection, cells were exposed to calcification media (DMEM with 10% fetal bovine serum, 100 units/ml penicillin, 100 ug/ml streptomycin, and 2mM sodium phosphate) for 10 days, changing media every 2-3 days to maintain phosphate levels. To assess amount of calcification, cells were stained with Alizarin Red (MilliporeSigma, Burlington, MA) for 60 seconds, then rinsed and imaged. Total number and area of calcifications was quantified by particle analysis using ImageJ 1.51s (NIH). Experiments were carried out in triplicate and as 3 independent experiments.

*Immunohistochemistry of human coronary and carotid arteries*

OCT-embedded, cryosectioned tissue samples from heart transplants (IRB protocol 802781) were thawed in TBS for 5min and fixed for 10min with 4%PFA (Electron Microscopy Services 15710). Non-specific antigens were blocked for 10min using Background Punisher (Biocare Medical BP974H). The sections were then incubated overnight in a humidity chamber with 1:200 TWIST1 rabbit polyclonal (Abcam ab50581) in DaVinci Green (Biocare Medical PD900H). Antibody detection was performed with a two-step, polymer based method using MACH3 anti-rabbit AP Polymer Detection reagent (Biocare Medical M3R533G). Aall wash steps were carried out with TBS. For chromogenic staining slides were incubated for 10min with Ferangi Blue (Biocare Medical FB813SBH). Counterstaining with Eosin (Sigma-Aldrich HT110216 ) was performed for 2min. Slides were then dehydrated for 1min in 90%EtOH, 1min in 100%EtOH and 2min in Histoclear (National Diagnostics HS-200) and embedded with Cytoseal XYL (Thermo Scientific 8312-4).

For staining of carotid plaques and normal tissues, all IHC reagents were from Biocare Medicals (Concord, CA). Tissues were fixed in 4% Zn-formaldehyde for 48 hours, dehydrated in 70% ethanol and embedded in paraffin blocks. Briefly, 5 μm sections were deparaffinized in Tissue Clear and rehydrated in gradually decreasing ethanol dilutions. For antigen retrieval, slides were subjected to high-pressure boiling in DIVA buffer (pH 6.0). After blocking with Background Sniper, TWIST1 and SMA primary antibodies (1:100 dilution, Abcam ab50581, and 1:1000 dilution Daco #M0851, respectively) , diluted in Da Vinci Green solution were applied on slides and incubated at room temperature for 1 hour. Isotype rabbit and mouse IgG were used as negative controls. For colocalisation staining, a double-stain probe-polymer system (Mach 2) containing alkaline phosphatase and horseradish peroxidase was applied, with subsequent detection using Warp Red and Vina Green. Slides were counterstained with Hematoxylin QS (Vector Laboratories, Burlingame, CA), dehydrated and mounted in Pertex (Histolab, Gothenburg, Sweden). Images were scanned using an automated SlideScaner system.

*In vitro differentiation of vascular smooth muscle cells and cytokine treatment*

Lineage-specific smooth muscle cells were generated as previously described [24]. Neural crest cells and neural crest derived SMCs were treated with 10 ng/ml IL1b over 6 hours.

*Microarray Hybridization and Analysis*

RNAs isolated from cells such as neural crest cells passage 2 and 7 (neural.crest_2, neural.crest_7) and SMCs derived from neural crest passage 2 and 7 (SMC_2, SMC_7) were hybridized to Illumina Human HT-12 BeadChip. To identify genes differentially expressed between the different microarray study groups, we used linear models for *limma* R-package. Hierarchical clustering was analysed and plotted with *Perseus* software, which used complete linkage and Euclidean distance metric to generate the heat map. In order to restrict the false discovery rate (FDR), Benjamini Hochberg FDR 0.05 was used as statistical differentially expressed genes.

*The BiKE study*

Patients undergoing surgery for symptomatic (S) or asymptomatic (AS), high-grade (>50% NASCET) carotid stenosis at the Department of Vascular Surgery, Karolinska University Hospital, Sweden were consecutively enrolled in the study and clinical data recorded on admission [25]. Symptoms of plaque instability were defined as transitory ischemic attack (TIA), minor stroke (MS) and amaurosis fugax (retinal TIA; AF). Patients without qualifying symptoms within 6 months prior to surgery were categorised as AS and indication for carotid endarterectomy based on results from the Asymptomatic Carotid Surgery Trial (ACST) [26]. Carotid endarterectomies (carotid plaques, CP) were collected at surgery and retained within the Biobank of Karolinska Endarterectomies (BiKE). This study involved 2 non-overlapping sub-cohorts of patients, where one Affymetrix microarray dataset was generated by profiling n=127 atherosclerotic plaques (of which n=87 were from S and 40 from AS patients) and n=10 normal arteries (further referred to as the ‘large dataset’) and the other by profiling n=50 plaques (n=41 from S and 9 from AS patients) and n=5 normal arteries (further referred as the ‘small dataset’). Normal artery controls (NA) were macroscopically disease-free iliac arteries and one aorta, obtained from organ donors without history of cardiovascular disease. Details of BiKE cohort patient demographics, sample processing and microarray analyses have been described previously [27-30]. Briefly, transcriptomic dataset analyses were performed with GraphPad Prism 6 and Bioconductor using a linear regression model adjusted for age and gender or a two-sided Student’s t-test assuming non-equal deviation, with correction for multiple comparisons according to Bonferroni. The microarray dataset is available from Gene Expression Omnibus (GSE21545). Tissue was also processed for histology and stained with Twist1 antibody (rabbit polyclonal, Abcam #50581). All samples were collected with informed consent from patients or organ donors’ guardians. All human studies were approved by the regional Ethical Committees.

References:

1. Anderson CA, Pettersson FH, Clarke GM, Cardon LR, Morris AP, Zondervan KT. Data quality control in genetic case-control association studies. Nat Protoc. 2010;5(9):1564-73. doi: 10.1038/nprot.2010.116. PubMed PMID: 21085122; PubMed Central PMCID: PMC3025522.

2. Genomes Project C, Auton A, Brooks LD, Durbin RM, Garrison EP, Kang HM, et al. A global reference for human genetic variation. Nature. 2015;526(7571):68-74. doi: 10.1038/nature15393. PubMed PMID: 26432245; PubMed Central PMCID: PMC4750478.

3. McCarthy S, Das S, Kretzschmar W, Delaneau O, Wood AR, Teumer A, et al. A reference panel of 64,976 haplotypes for genotype imputation. Nat Genet. 2016;48(10):1279-83. doi: 10.1038/ng.3643. PubMed PMID: 27548312; PubMed Central PMCID: PMC5388176.

4. Loh PR, Danecek P, Palamara PF, Fuchsberger C, Y AR, H KF, et al. Reference-based phasing using the Haplotype Reference Consortium panel. Nat Genet. 2016;48(11):1443-8. doi: 10.1038/ng.3679. PubMed PMID: 27694958; PubMed Central PMCID: PMC5096458.

5. Durbin R. Efficient haplotype matching and storage using the positional Burrows-Wheeler transform (PBWT). Bioinformatics. 2014;30(9):1266-72. doi: 10.1093/bioinformatics/btu014. PubMed PMID: 24413527; PubMed Central PMCID: PMC3998136.

6. Vaquero-Garcia J, Barrera A, Gazzara MR, Gonzalez-Vallinas J, Lahens NF, Hogenesch JB, et al. A new view of transcriptome complexity and regulation through the lens of local splicing variations. Elife. 2016;5:e11752. Epub 2016/02/02. doi: 10.7554/eLife.11752. PubMed PMID: 26829591; PubMed Central PMCID: PMCPMC4801060.

7. Li YI, van de Geijn B, Raj A, Knowles DA, Petti AA, Golan D, et al. RNA splicing is a primary link between genetic variation and disease. Science. 2016;352(6285):600-4. Epub 2016/04/30. doi: 10.1126/science.aad9417. PubMed PMID: 27126046; PubMed Central PMCID: PMCPMC5182069.

8. Gormley P, Anttila V, Winsvold BS, Palta P, Esko T, Pers TH, et al. Meta-analysis of 375,000 individuals identifies 38 susceptibility loci for migraine. Nat Genet. 2016;48(8):856-66. doi: 10.1038/ng.3598. PubMed PMID: 27322543; PubMed Central PMCID: PMC5331903.

9. Jones GT, Tromp G, Kuivaniemi H, Gretarsdottir S, Baas AF, Giusti B, et al. Meta-Analysis of Genome-Wide Association Studies for Abdominal Aortic Aneurysm Identifies Four New Disease-Specific Risk Loci. Circ Res. 2017;120(2):341-53. Epub 2016/12/03. doi: 10.1161/CIRCRESAHA.116.308765. PubMed PMID: 27899403; PubMed Central PMCID: PMCPMC5253231.

10. Traylor M, Farrall M, Holliday EG, Sudlow C, Hopewell JC, Cheng YC, et al. Genetic risk factors for ischaemic stroke and its subtypes (the METASTROKE collaboration): a meta-analysis of genome-wide association studies. Lancet Neurol. 2012;11(11):951-62. doi: 10.1016/S1474-4422(12)70234-X. PubMed PMID: 23041239; PubMed Central PMCID: PMC3490334.

11. Harvey CT, Moyerbrailean GA, Davis GO, Wen X, Luca F, Pique-Regi R. QuASAR: quantitative allele-specific analysis of reads. Bioinformatics. 2015;31(8):1235-42. doi: 10.1093/bioinformatics/btu802. PubMed PMID: 25480375; PubMed Central PMCID: PMC4393517.

12. Li H, Handsaker B, Wysoker A, Fennell T, Ruan J, Homer N, et al. The Sequence Alignment/Map format and SAMtools. Bioinformatics. 2009;25(16):2078-9. Epub 2009/06/10. doi: 10.1093/bioinformatics/btp352. PubMed PMID: 19505943; PubMed Central PMCID: PMCPMC2723002.

13. Storey JD, Tibshirani R. Statistical significance for genomewide studies. Proc Natl Acad Sci U S A. 2003;100(16):9440-5. Epub 2003/07/29. doi: 10.1073/pnas.1530509100. PubMed PMID: 12883005; PubMed Central PMCID: PMCPMC170937.

14. Carithers LJ, Ardlie K, Barcus M, Branton PA, Britton A, Buia SA, et al. A Novel Approach to High-Quality Postmortem Tissue Procurement: The GTEx Project. Biopreserv Biobank. 2015;13(5):311-9. doi: 10.1089/bio.2015.0032. PubMed PMID: 26484571; PubMed Central PMCID: PMC4675181.

15. Giambartolomei C, Vukcevic D, Schadt EE, Franke L, Hingorani AD, Wallace C, et al. Bayesian test for colocalisation between pairs of genetic association studies using summary statistics. PLoS Genet. 2014;10(5):e1004383. doi: 10.1371/journal.pgen.1004383. PubMed PMID: 24830394; PubMed Central PMCID: PMC4022491.

16. Nikpay M, Goel A, Won HH, Hall LM, Willenborg C, Kanoni S, et al. A comprehensive 1,000 Genomes-based genome-wide association meta-analysis of coronary artery disease. Nat Genet. 2015;47(10):1121-30. doi: 10.1038/ng.3396. PubMed PMID: 26343387; PubMed Central PMCID: PMC4589895.

17. Jones GT, Tromp G, Kuivaniemi H, Gretarsdottir S, Baas AF, Giusti B, et al. Meta-Analysis of Genome-Wide Association Studies for Abdominal Aortic Aneurysm Identifies Four New Disease-Specific Risk Loci. Circ Res. 2016. doi: 10.1161/CIRCRESAHA.116.308765. PubMed PMID: 27899403.

18. Yang J, Ferreira T, Morris AP, Medland SE, Genetic Investigation of ATC, Replication DIG, et al. Conditional and joint multiple-SNP analysis of GWAS summary statistics identifies additional variants influencing complex traits. Nat Genet. 2012;44(4):369-75, S1-3. doi: 10.1038/ng.2213. PubMed PMID: 22426310; PubMed Central PMCID: PMC3593158.

19. Kuroda K, Tani S, Tamura K, Minoguchi S, Kurooka H, Honjo T. Delta-induced Notch signaling mediated by RBP-J inhibits MyoD expression and myogenesis. J Biol Chem. 1999;274(11):7238-44. PubMed PMID: 10066785.

20. Minoguchi S, Taniguchi Y, Kato H, Okazaki T, Strobl LJ, Zimber-Strobl U, et al. RBP-L, a transcription factor related to RBP-Jkappa. Mol Cell Biol. 1997;17(5):2679-87. PubMed PMID: 9111338; PubMed Central PMCID: PMC232118.

21. Nurnberg ST, Rendon A, Smethurst PA, Paul DS, Voss K, Thon JN, et al. A GWAS sequence variant for platelet volume marks an alternative DNM3 promoter in megakaryocytes near a MEIS1 binding site. Blood. 2012;120(24):4859-68. doi: 10.1182/blood-2012-01-401893. PubMed PMID: 22972982; PubMed Central PMCID: PMC3520622.

22. Thakore PI, D'Ippolito AM, Song L, Safi A, Shivakumar NK, Kabadi AM, et al. Highly specific epigenome editing by CRISPR-Cas9 repressors for silencing of distal regulatory elements. Nat Methods. 2015;12(12):1143-9. Epub 2015/10/27. doi: 10.1038/nmeth.3630. PubMed PMID: 26501517; PubMed Central PMCID: PMCPMC4666778.

23. Zhao Q, Wirka R, Nguyen T, Nagao M, Cheng P, Miller CL, et al. TCF21 and AP-1 interact through epigenetic modifications to regulate coronary artery disease gene expression. Genome Med. 2019;11(1):23. Epub 2019/04/25. doi: 10.1186/s13073-019-0635-9. PubMed PMID: 31014396; PubMed Central PMCID: PMCPMC6480881.

24. Cheung C, Bernardo AS, Trotter MW, Pedersen RA, Sinha S. Generation of human vascular smooth muscle subtypes provides insight into embryological origin-dependent disease susceptibility. Nat Biotechnol. 2012;30(2):165-73. doi: 10.1038/nbt.2107. PubMed PMID: 22252507; PubMed Central PMCID: PMC3272383.

25. Rothwell PM, Pendlebury ST, Wardlaw J, Warlow CP. Critical appraisal of the design and reporting of studies of imaging and measurement of carotid stenosis. Stroke. 2000;31(6):1444-50. PubMed PMID: 10835470.

26. Halliday A, Harrison M, Hayter E, Kong X, Mansfield A, Marro J, et al. 10-year stroke prevention after successful carotid endarterectomy for asymptomatic stenosis (ACST-1): a multicentre randomised trial. Lancet. 2010;376(9746):1074-84. doi: 10.1016/S0140-6736(10)61197-X. PubMed PMID: 20870099; PubMed Central PMCID: PMC2956884.

27. Folkersen L, Persson J, Ekstrand J, Agardh HE, Hansson GK, Gabrielsen A, et al. Prediction of ischemic events on the basis of transcriptomic and genomic profiling in patients undergoing carotid endarterectomy. Mol Med. 2012;18:669-75. doi: 10.2119/molmed.2011.00479. PubMed PMID: 22371308; PubMed Central PMCID: PMC3388132.

28. Razuvaev A, Ekstrand J, Folkersen L, Agardh H, Markus D, Swedenborg J, et al. Correlations between clinical variables and gene-expression profiles in carotid plaque instability. Eur J Vasc Endovasc Surg. 2011;42(6):722-30. doi: 10.1016/j.ejvs.2011.05.023. PubMed PMID: 21741279.

29. Perisic L, Hedin E, Razuvaev A, Lengquist M, Osterholm C, Folkersen L, et al. Profiling of atherosclerotic lesions by gene and tissue microarrays reveals PCSK6 as a novel protease in unstable carotid atherosclerosis. Arterioscler Thromb Vasc Biol. 2013;33(10):2432-43. doi: 10.1161/ATVBAHA.113.301743. PubMed PMID: 23908247.

30. Perisic L, Aldi S, Sun Y, Folkersen L, Razuvaev A, Roy J, et al. Gene expression signatures, pathways and networks in carotid atherosclerosis. J Intern Med. 2016;279(3):293-308. doi: 10.1111/joim.12448. PubMed PMID: 26620734.
