# Supplementary figures and images for: Genomic profiling of human vascular cells identifies TWIST1 as a causal gene for common vascular diseases
Source: PLoS Genet. 2020 Jan 9;16(1):e1008538. doi: 10.1371/journal.pgen.1008538 (PMC6975560; doi:10.1371/journal.pgen.1008538)

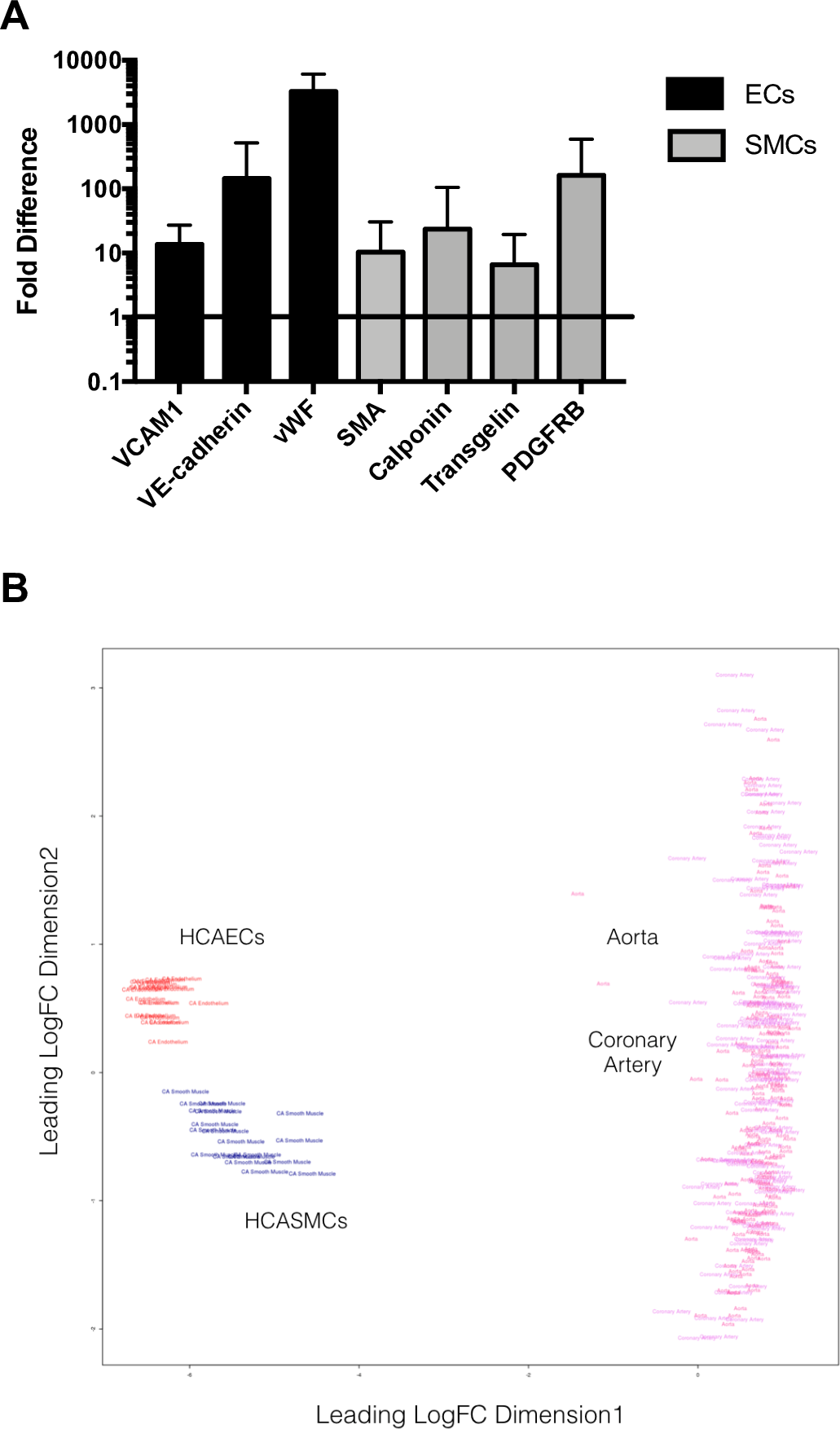

Supplement: S1 Fig — A) Fold difference in expression of endothelial cell markers and smooth muscle cell markers from the RNASeq data in 19 paired samples. Endothelial genes are presented in black as the ratio of TPMs in HCAECs versus HCSMCs, and Smooth muscle genes are presented in grey as the ratio of TPMs in HCASMCs to HCAECs. B) Multi-dimensional scaling plot of the 500 most differentially expressed genes from GTEX RNA-Seq data from 205 aortic (pink) and 117 coronary artery (violet) samples as well as 19/20 in vitro cultured HCASMC (blue) and HCAEC (red) samples reveals significant overlap between human aortic and coronary artery tissues. HCAECs and HCSMCs cluster separately from each other with ECs showing tighter clustering among samples. Both types of primary cell lines cluster separately from arterial tissue, which may be due to the artificial nature of the in vitro environment. (TIF) [file pgen.1008538.s004.tif]

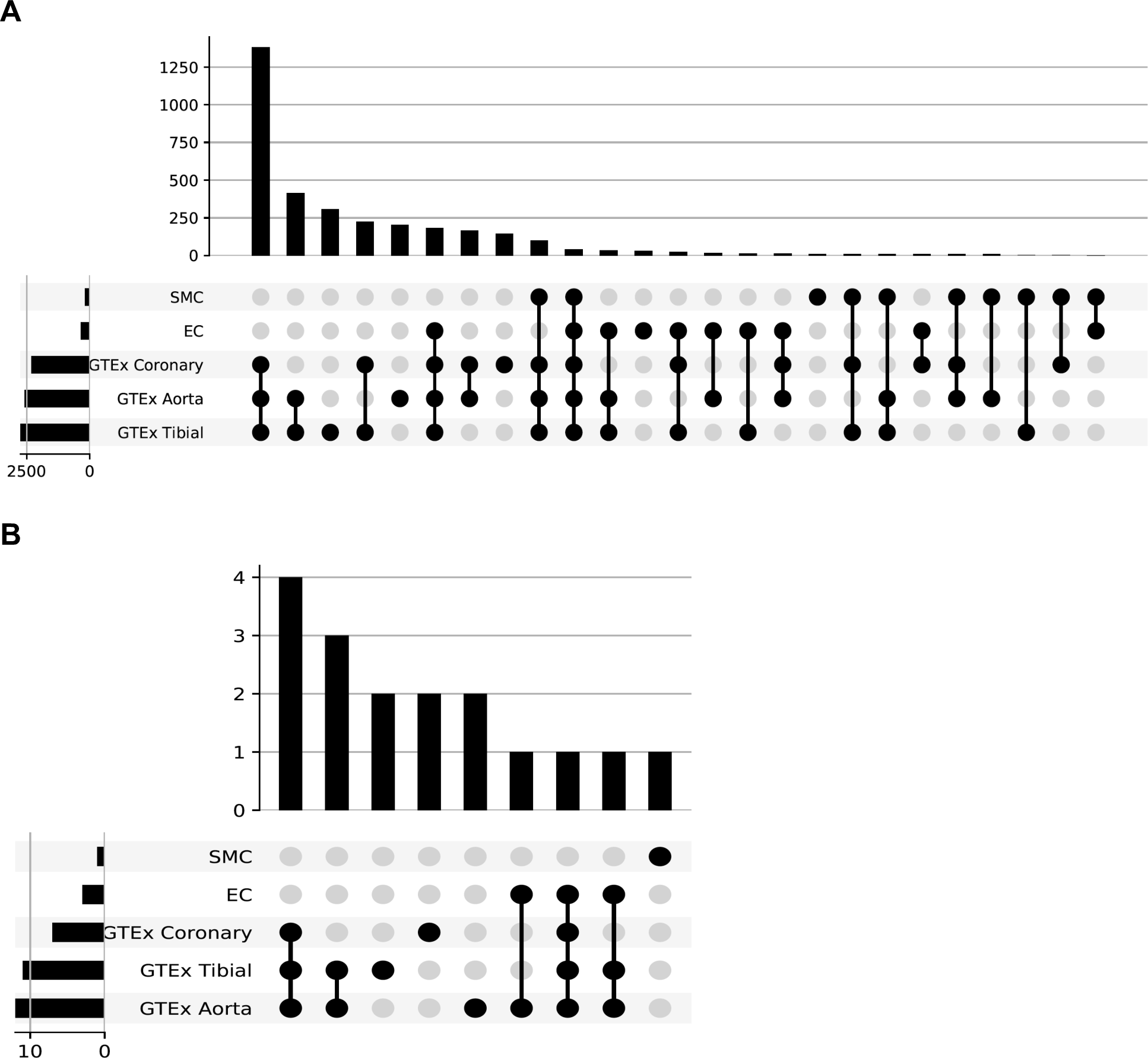

Supplement: S2 Fig — A) UpSet plot of genes with sQTLs in the HCSMC (SMC), HCAEC (EC) and GTEx datasets (FDR < 0.05, regression test). Vertical bars represent the count of unique genes per set. Below the bar graphs, each dot represents a dataset and intersecting sets are represented by lines connecting dots. Horizontal bars represent the total number of genes with putative sQTLs in each dataset. B) UpSet plot of all genes with putative sQTLs in the HCASMC/HCAEC and GTEx cohorts that colocalize with any signal for association with cardiovascular disease. (TIF) [file pgen.1008538.s005.tif]

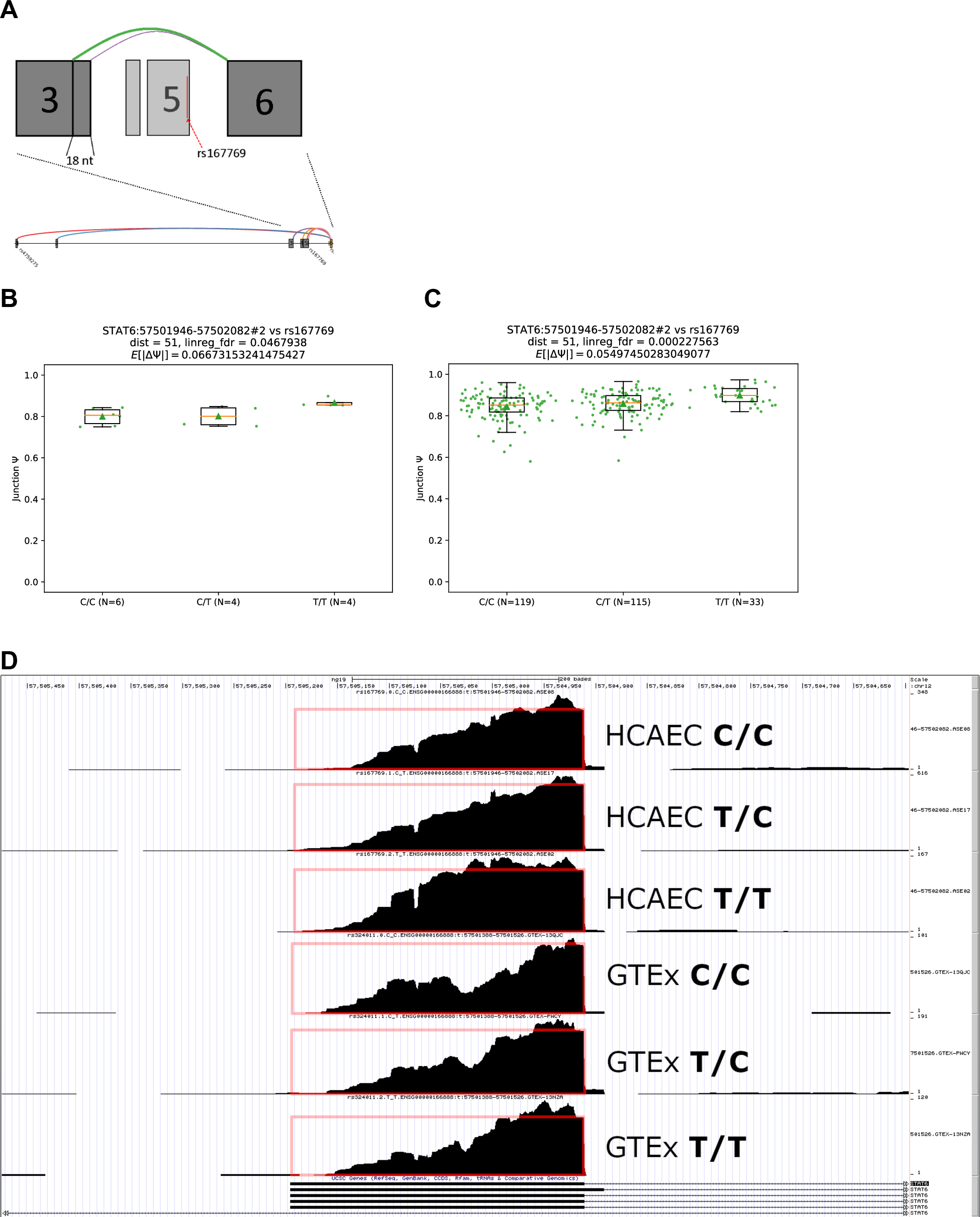

Supplement: S3 Fig — A) Splicegraph structure of STAT6 near the 5’ end, showing the implicated LSV targeting exon 6. Inset zooms in on the relevant exons and splice junctions (not to scale). B)—C) Scatterbox plots of PSI for the alternative 5’ splice site event in STAT6 exon 3, which is the first exon in the majority of transcripts of STAT6, using data from HCAEC and GTEx, respectively. Each plot represents samples of the indicated genotype at rs167769. Each green point represent inclusion level (PSI) quantified in a sample of the LSV’s green junction in A. D) RNA-seq reads mapping to the alternative 5’ splice site event at the canonical first exon of STAT6 (purple and green junctions in A). Tracks are labeled with the dataset of origin and sample genotype at rs167769 (HCAEC) or rs324011 (GTEx coronary artery). Representative samples were randomly selected from the pool of all samples with the indicated genotype in their respective dataset. Reads mapping into the canonical exon body are outlined in a red box. Reads mapping to the 18-nt extension are immediately to the right of this box. The UCSC transcript annotation track is depicted on the bottom for reference; the bottommost transcript uses a different first exon not depicted. (TIF) [file pgen.1008538.s006.tif]

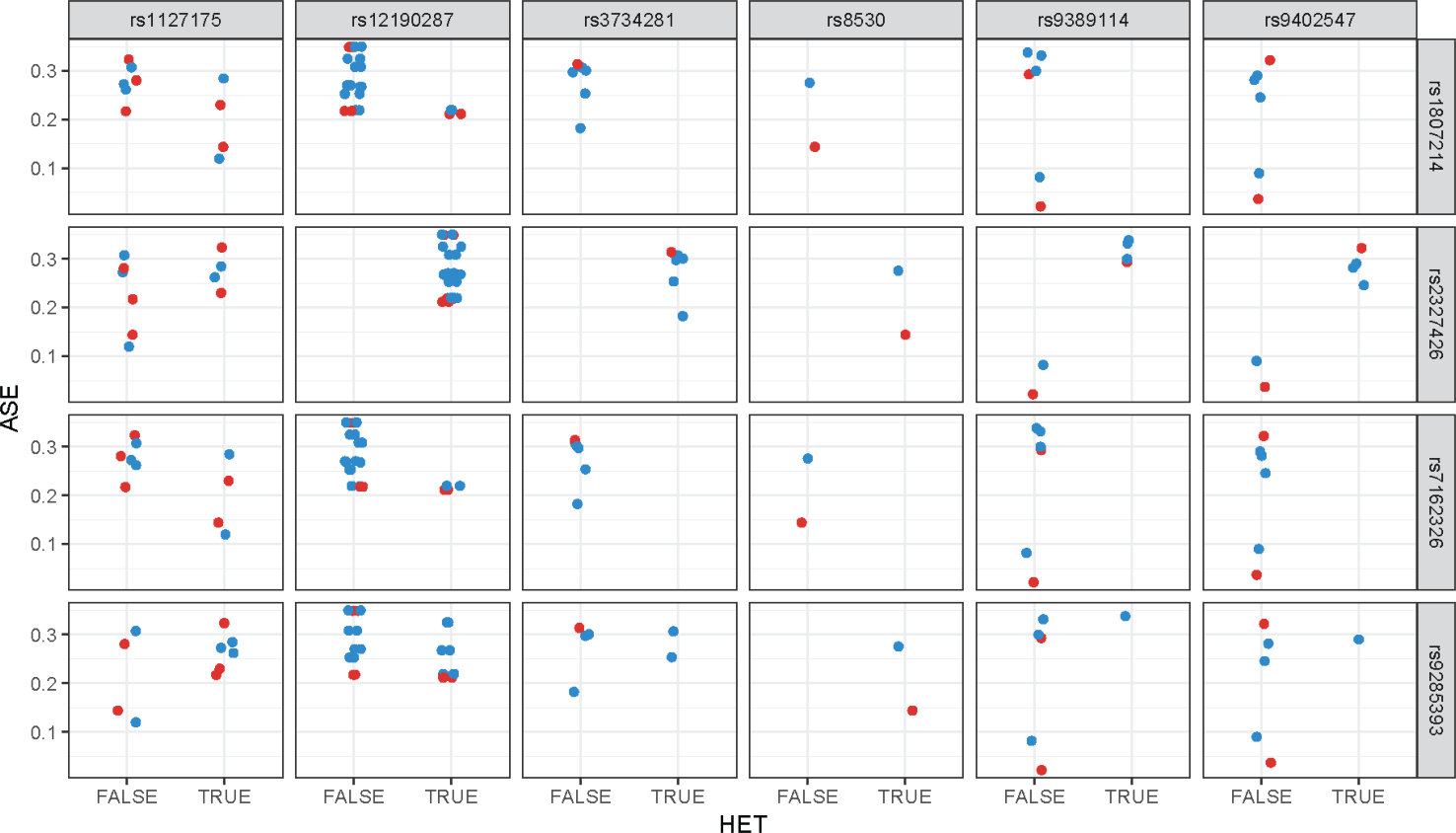

Supplement: S4 Fig — (TIF) [file pgen.1008538.s007.tif]

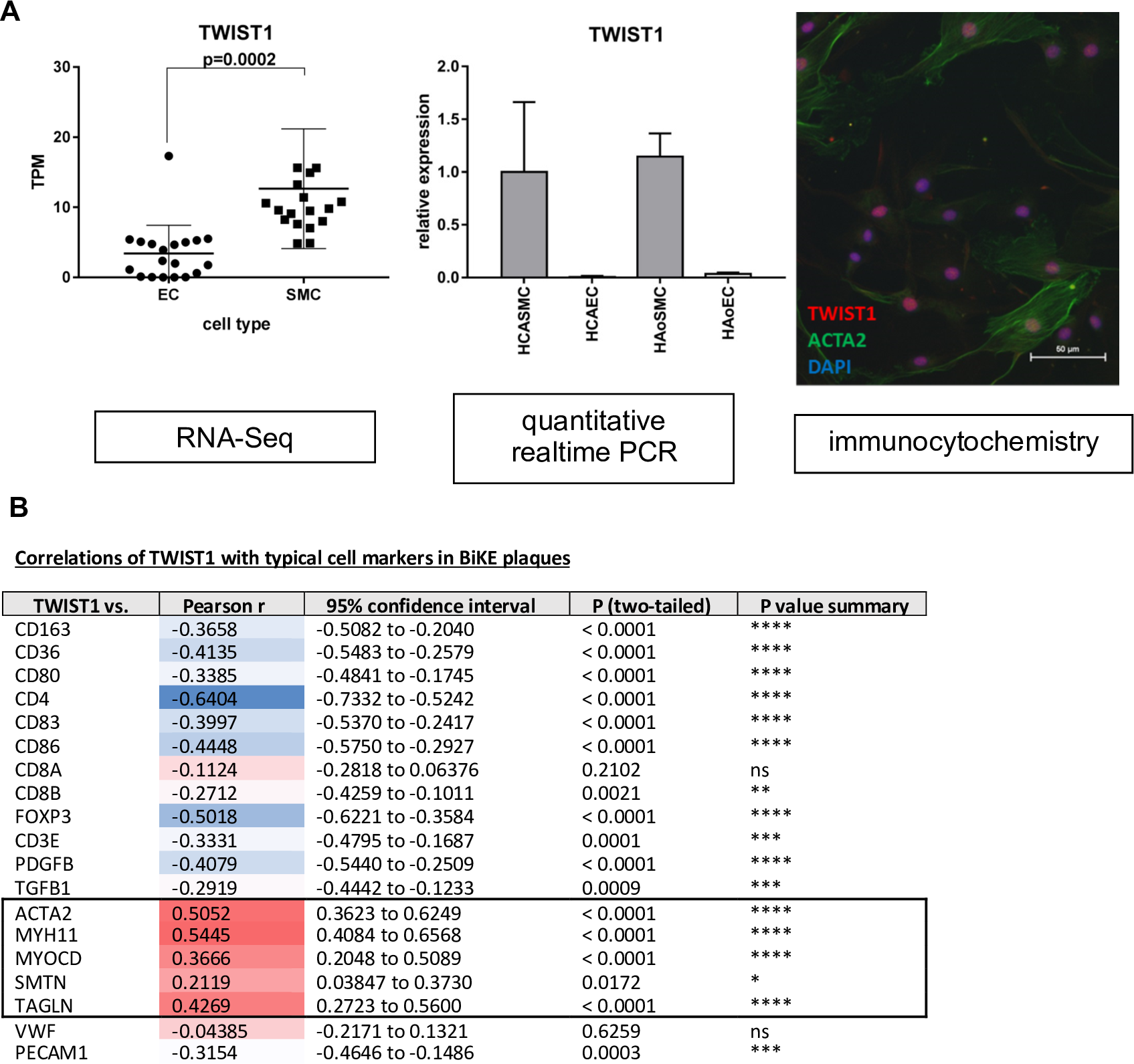

Supplement: S5 Fig — A) TWIST1 is increased in SMCs relative ECs based on both RNASeq data and qRTPCR. Immunocytochemistry shows nuclear TWIST1 staining in ACTA2-positive SMCs. B) Expression of TWIST1 is positively correlated with SMC markers (red) and negatively correlated with EC and immune cell markers (blue) in human atherosclerotic plaque samples from the BiKE study. (TIF) [file pgen.1008538.s008.tif]

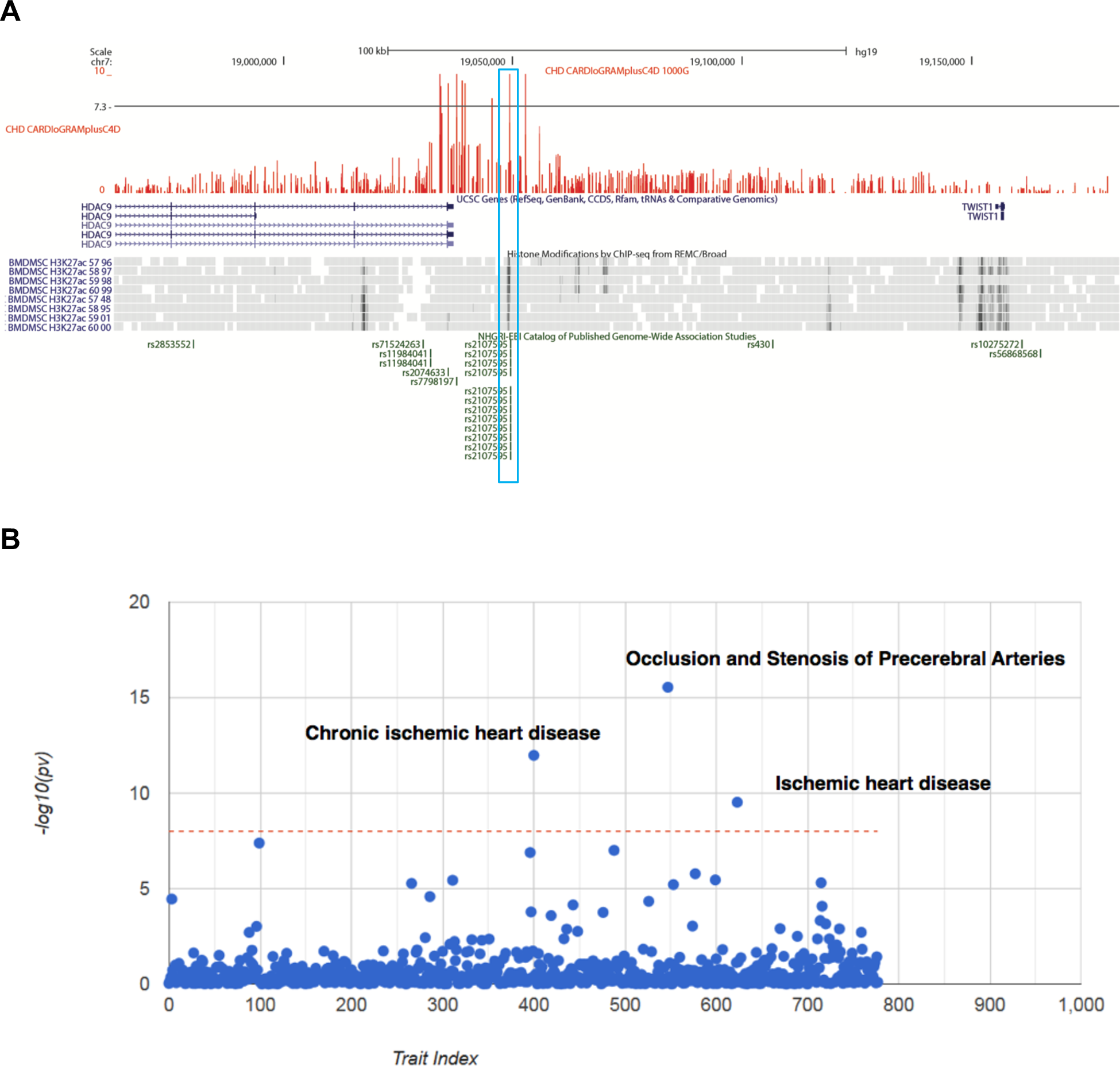

Supplement: S6 Fig — A) UCSC Browser view displaying the genomic landscape around GWAS SNP rs2107595 (box). H3K27ac histone modification ChIP-Seq data from bone-marrow derived mesenchymal stem cells from the ENCODE project is also displayed. There is high H3K27ac at this locus which indicates that this area is likely an active enhancer. B) Phenome-Wide Association Study data from the UK Biobank shows that rs2107595 is significantly associated with three common vascular disease phenotypes. (TIF) [file pgen.1008538.s009.tif]

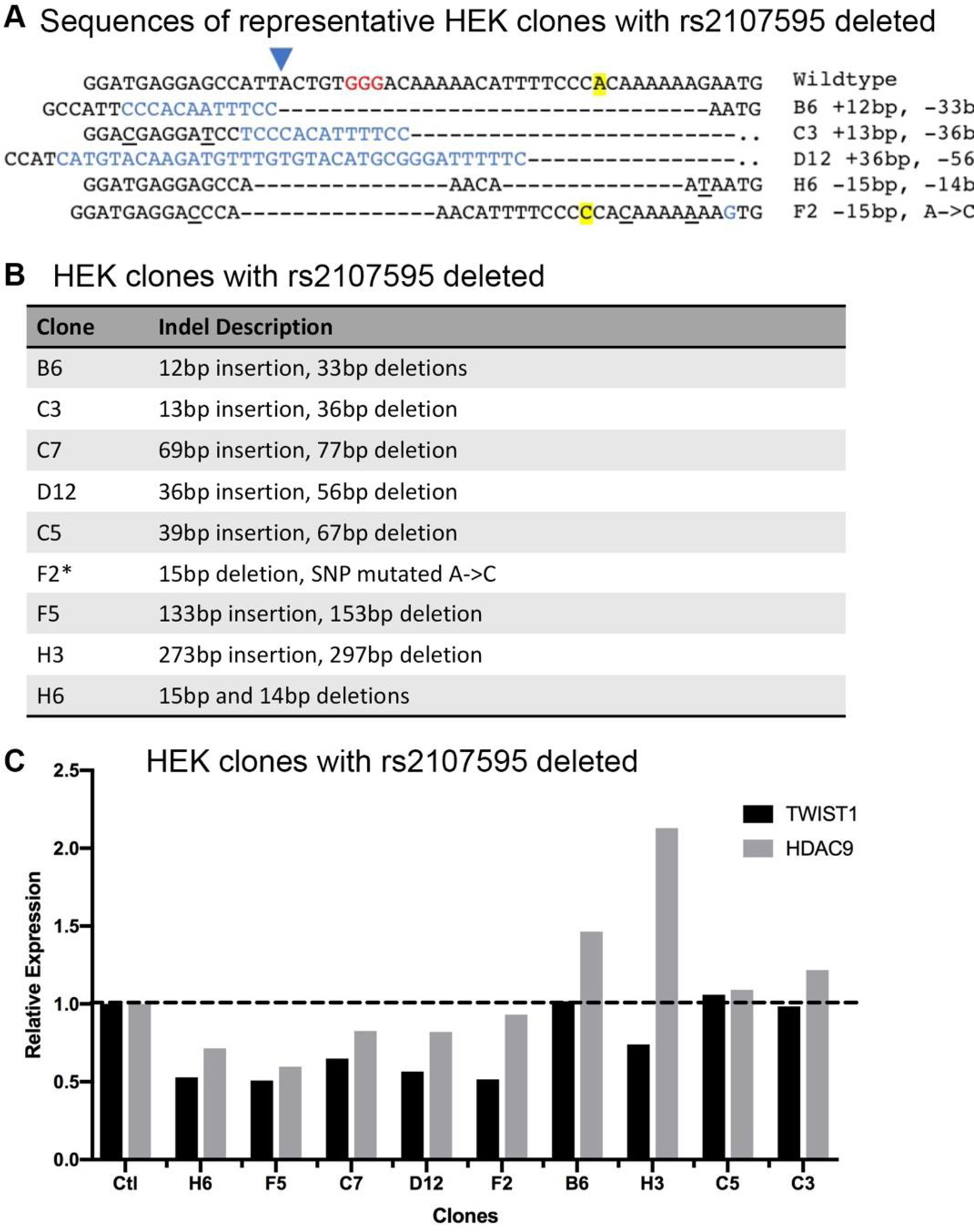

Supplement: S7 Fig — A) The wildtype sequence shows rs2107595 (yellow), PAM sequence (red) and the cut site within the guide RNA sequence (blue arrowhead). Representative sequences for editted clones are aligned below to show the effects of CRISPR/Cas9 editing in this genomic region. B) A summary of all clones generated and used for analysis show a range of insertion/deletions. In all cases, rs2107595 is either missing or editted (as in clone F2). C) TWIST1 and HDAC9 gene expression is shown for each cell line relative to control. Disruption of rs2107595 decreased TWIST1 expression in most cell lines. (TIF) [file pgen.1008538.s010.tif]

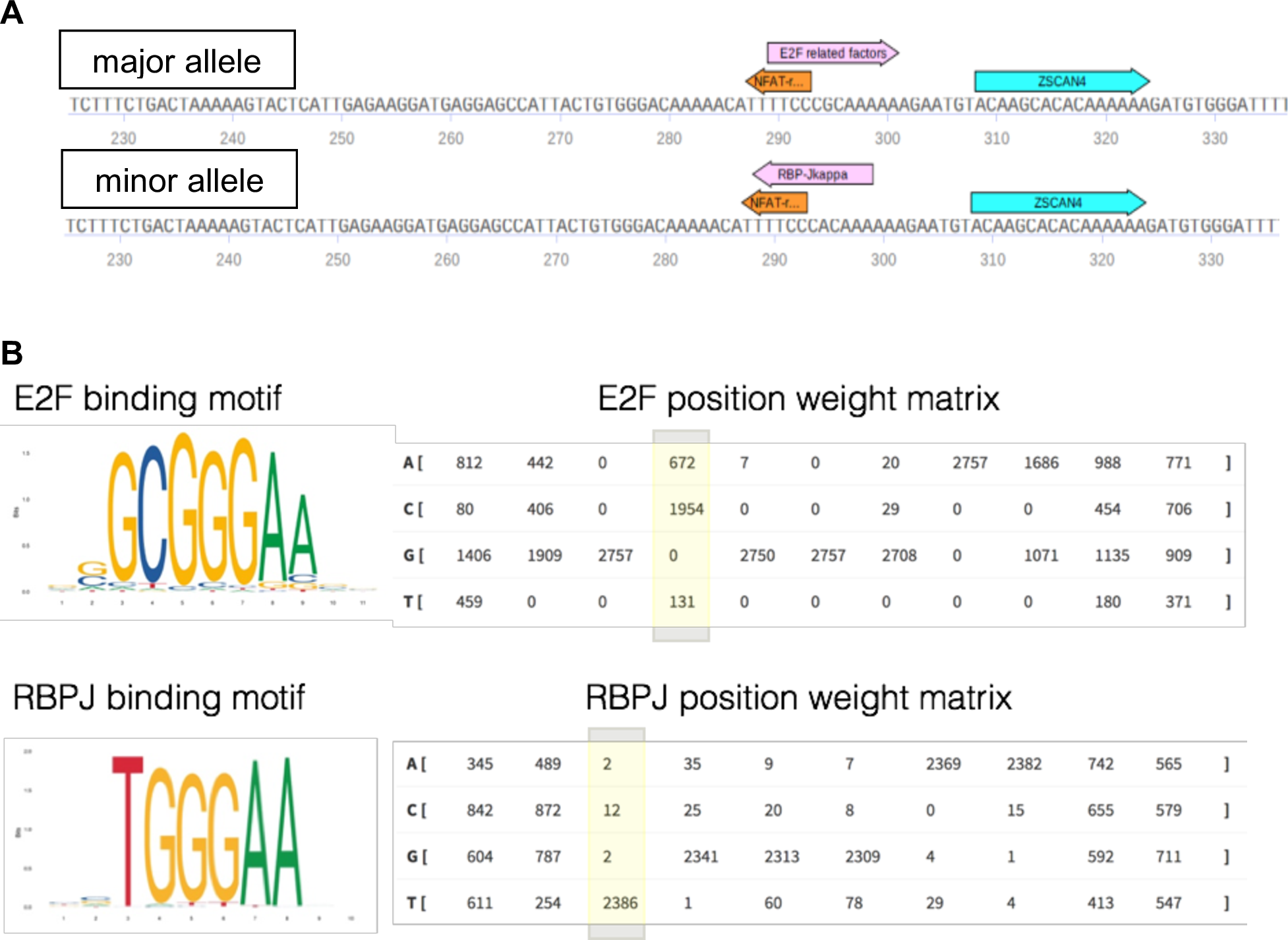

Supplement: S8 Fig — A) Overview of proposed transcription factor binding to the major and minor alleles based on Transfact Professional (2014.4 data release). B) Using JASPAR, an open-access database of transcription factor binding profiles, we find that if there is a G at the rs2107595 locus (and C on the complementary strand), this base pair forms part of an E2F binding motif. The E2F position matrix shows that if this is converted to a T, E2F will bind ~ 5% of the time (top yellow box). When the minor allele is present, and there is a T on the complementary strand of the locus, this base pair forms part of an RBPJ binding motif. The corresponding RBPJ position weight matrix shows that a C at this position would result in RBPJ binding ~ 0.5% of the time (bottom yellow box). (TIF) [file pgen.1008538.s011.tif]

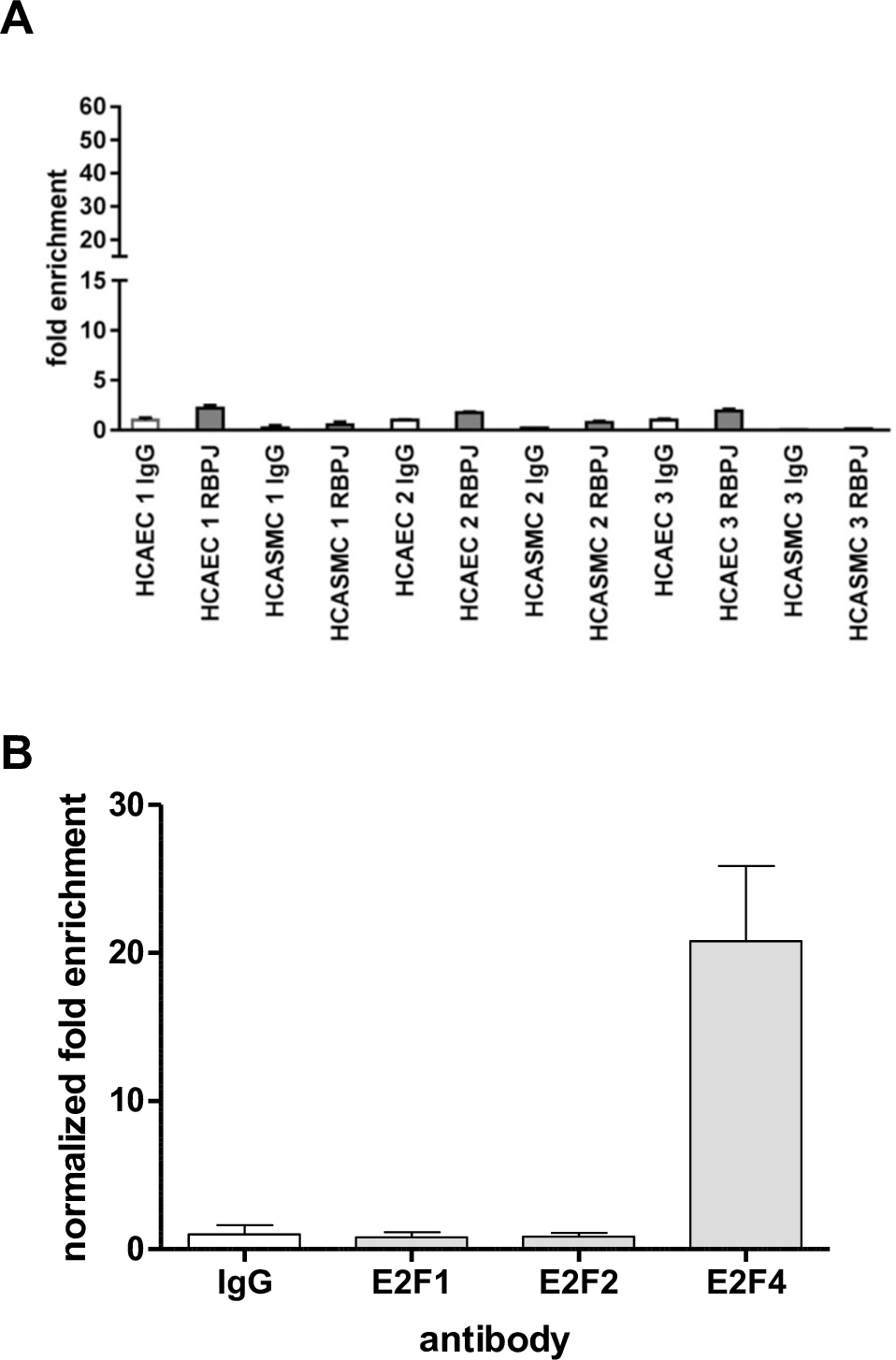

Supplement: S9 Fig — A) ChIP was performed using RBPJ antibody and IgG controls on heterozygous HCASMCs and HCAECs. A region 17kb upstream of rs2107595 was amplified to serve as negative control. As expected, there was no significant enrichment of this area with RBPJ relative to IgG control. B) ChIP was performed using three different E2F antibodies (E2F1, E2F2, E2F4) as well as IgG control on heterozygous HCSMCs. qPCR of the region around rs2107595 shows significant enrichment with E2F4 suggesting E2F4, but not E2F1 or E2F2, binds this region. (TIF) [file pgen.1008538.s012.tif]

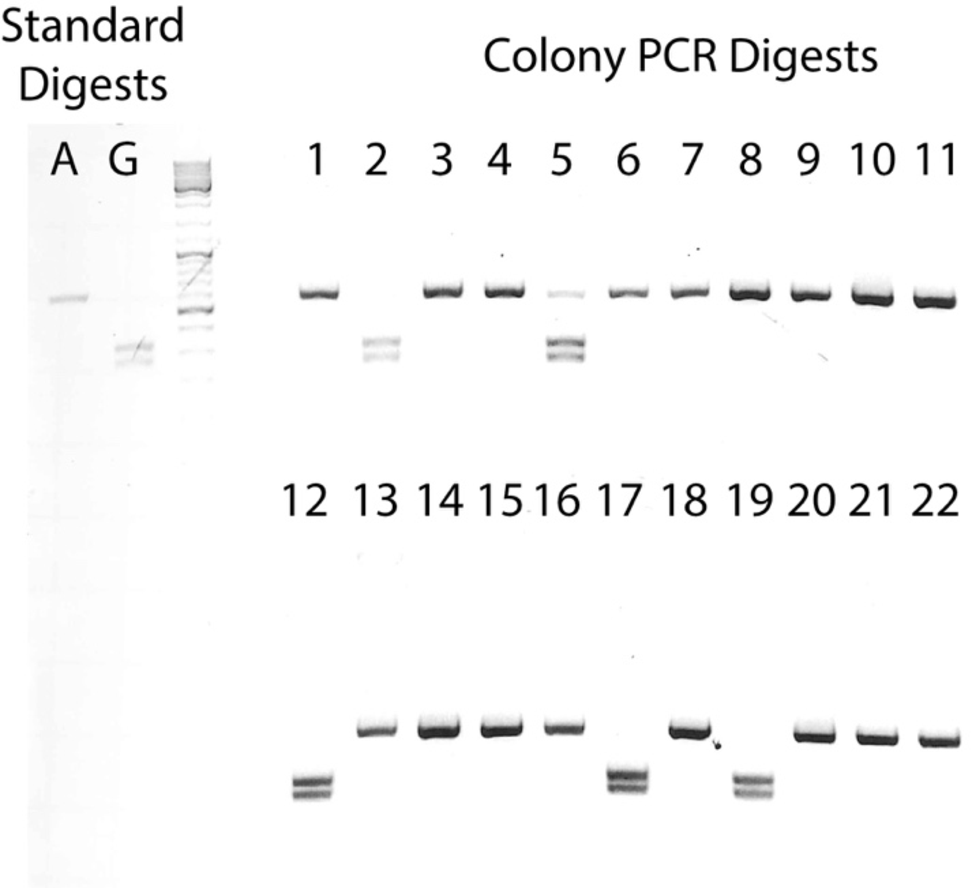

Supplement: S10 Fig — Chromatin-Immunoprecipitation was performed using RBPJ on heterozygous HCASMCs. The region surrounding rs2107595 was amplified and cloned into sequencing vectors. PCR digestion of 22 colonies show that RBPJ is preferentially binding the risk (A) allele (17/22 colonies). (TIF) [file pgen.1008538.s013.tif]

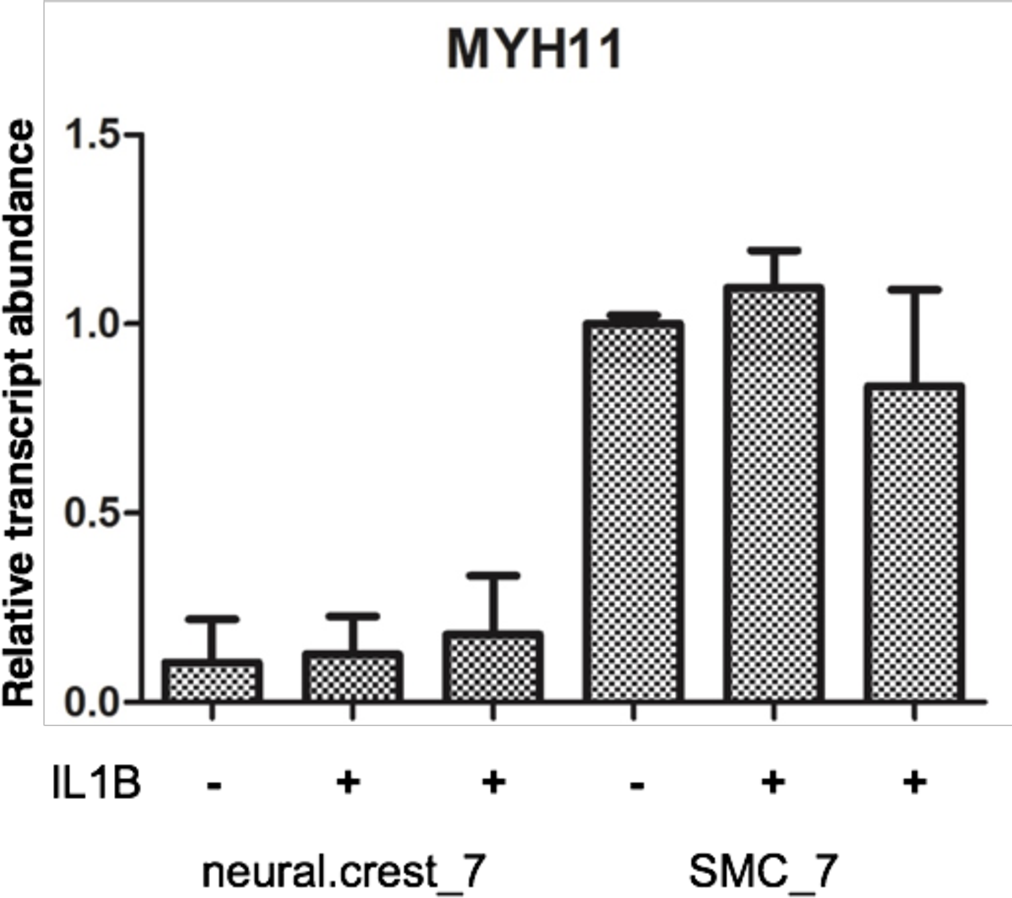

Supplement: S11 Fig — Progenitor cells express less MYH11 than derived SMC cells, and that MYH11 does not increase in response to IL1B. (TIF) [file pgen.1008538.s014.tif]

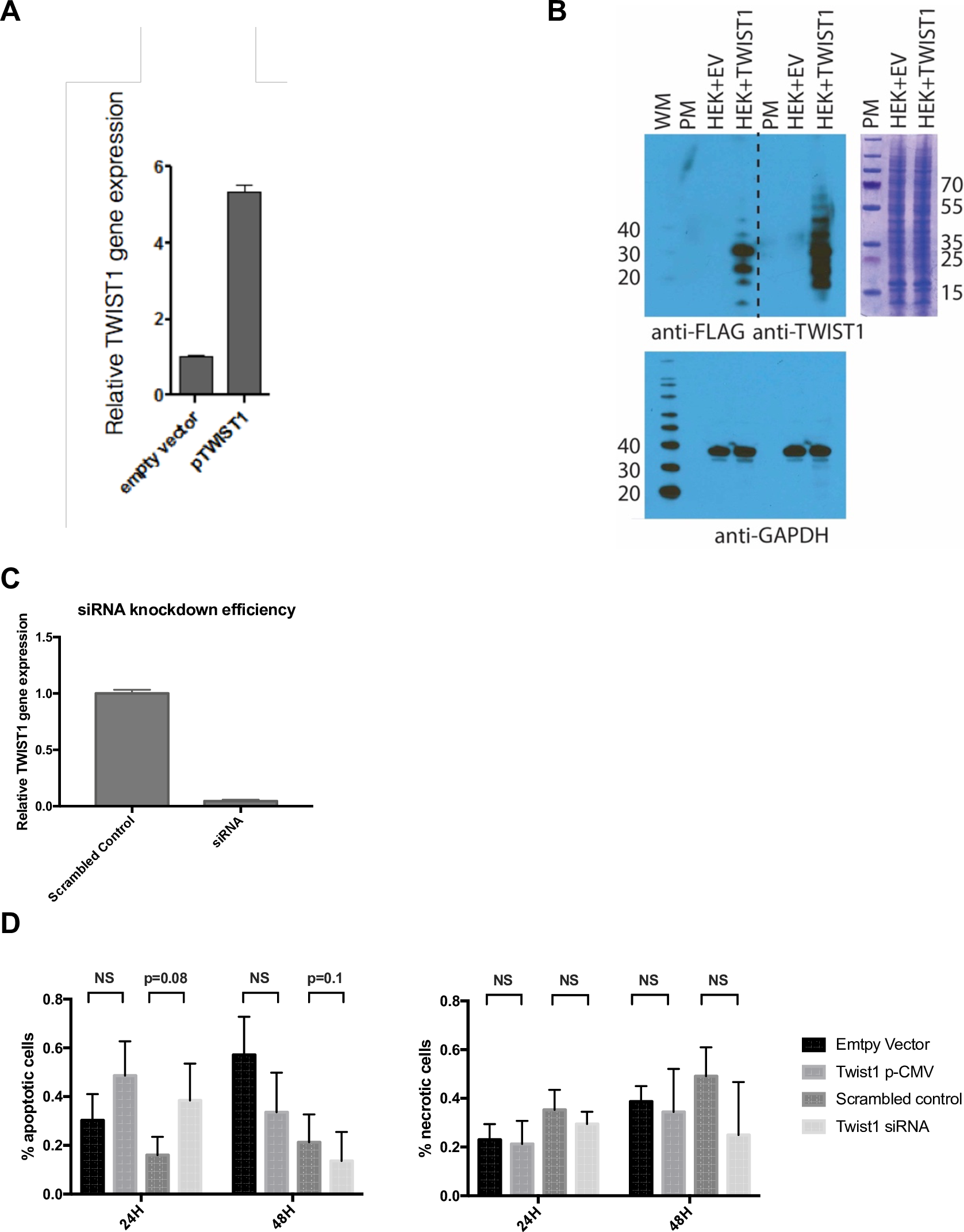

Supplement: S12 Fig — A) CMV6-promoter driven TWIST1 overexpression ((pTWIST1) resulted in a ~ 5-fold increase in TWIST1 gene expression. B) TWIST1 protein is increased as well in HEK293T cells. WM: Western Marker, PM: Prestained Marker. C) SiRNA knockdown of TWIST1 resulted in ~95% decrease in TWIST1 gene expression. D) TWIST1 overexpression and knockdown at 24H and 48H had no consistent effect on apoptosis or necrosis. (TIF) [file pgen.1008538.s015.tif]

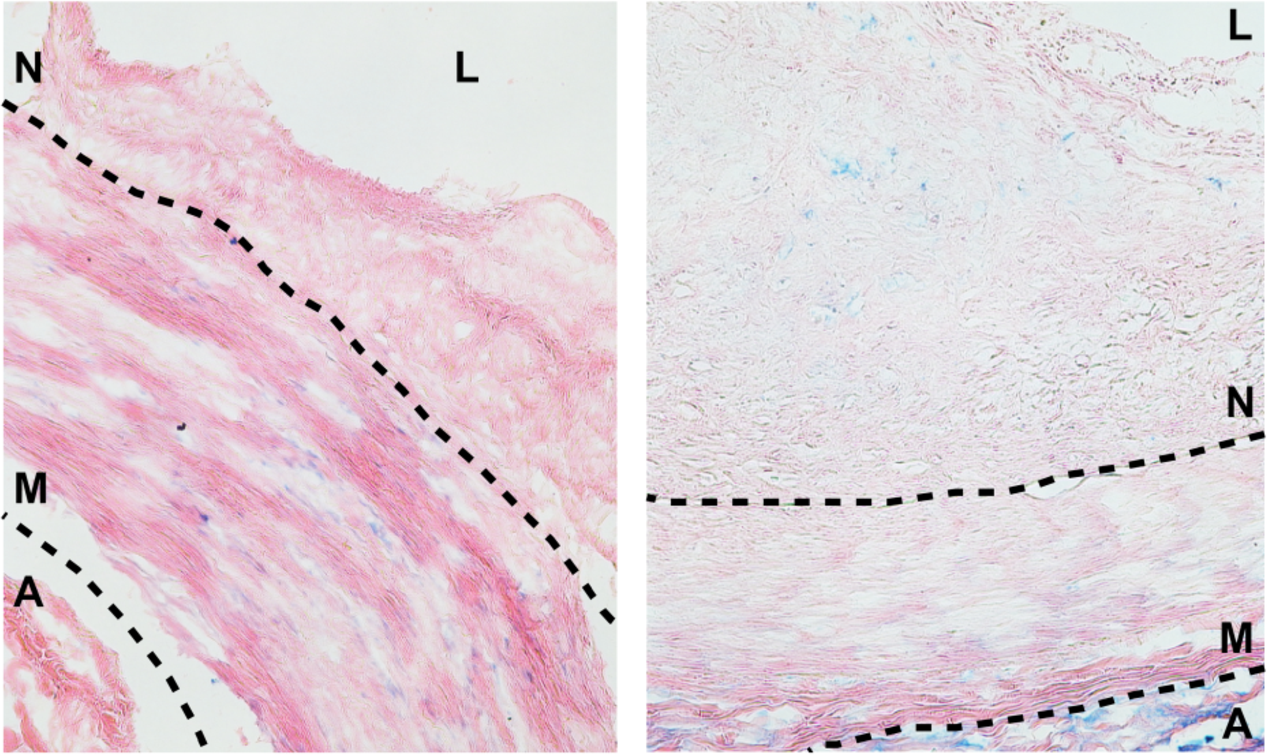

Supplement: S13 Fig — TWIST1 protein (blue) is present in the media (M) and in the plaque. L = Lumen; N = Neointima, M = Media; A = Adventitia. (TIF) [file pgen.1008538.s016.tif]

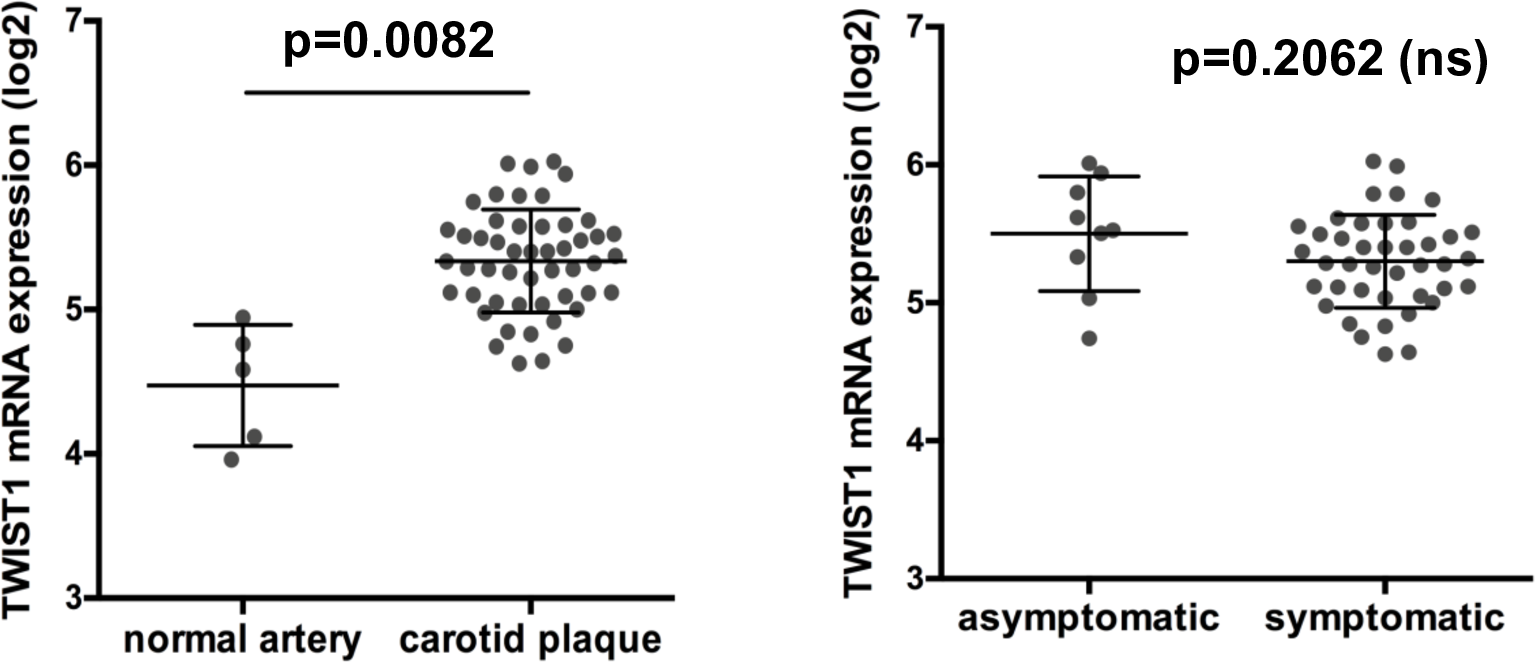

Supplement: S14 Fig — A) Twist1 is higher in carotid plaques relative to normal arteries. B) There is a trend towards decreased Twist1 expression in symptomatic lesions relative to asymptomatic lesions. (TIF) [file pgen.1008538.s017.tif]
